# Cord blood metabolic signatures predictive of childhood overweight and rapid growth

**DOI:** 10.1038/s41366-021-00888-1

**Published:** 2021-07-12

**Authors:** Evangelos Handakas, Pekka Keski-Rahkonen, Lida Chatzi, Rossella Alfano, Theano Roumeliotaki, Michelle Plusquin, Léa Maitre, Lorenzo Richiardi, Sonia Brescianini, Augustin Scalbert, Nivonirina Robinot, Tim Nawrot, Franco Sassi, Martine Vrijheid, Paolo Vineis, Oliver Robinson

**Affiliations:** 1 Μedical Research Council Centre for Environment and Health, School of Public Health, Imperial College London, London, UK; 2 Nutrition and Metabolism Section, International Agency for Research on Cancer, Lyon, France; 3 Department of Preventive Medicine, Keck School of Medicine, University of Southern California, Los Angeles, CA USA; 4 Centre for Environmental Sciences, Hasselt University, Diepenbeek, Belgium; 5 Department of Social Medicine, Faculty of Medicine, University of Crete, Heraklion, Greece; 6 Barcelona Institute of Global Health (ISGlobal), Barcelona, Spain; 7 Universitat Pompeu Fabra, Barcelona, Spain; 8 CIBER Epidemiología y Salud Pública (CIBERESP), Madrid, Spain; 9 Cancer Epidemiology Unit, Department of Medical Sciences, University of Turin and CPO‐Piemonte, Torino, Italy; 10 Centre for Behavioural Science and Mental Health, Istituto Superiore di Sanità, Rome, Italy; 11 Centre for Health Economics & Policy Innovation, Department of Economics & Public Policy, Imperial College Business School, South Kensington Campus, London, UK

**Keywords:** Risk factors, Biochemistry, Chemical biology, Obesity, Epidemiology

## Abstract

**Introduction:**

Metabolomics may identify biological pathways predisposing children to the risk of overweight and obesity. In this study, we have investigated the cord blood metabolic signatures of rapid growth in infancy and overweight in early childhood in four European birth cohorts.

**Methods:**

Untargeted liquid chromatography-mass spectrometry metabolomic profiles were measured in cord blood from 399 newborns from four European cohorts (ENVIRONAGE, Rhea, INMA and Piccolipiu). Rapid growth in the first year of life and overweight in childhood was defined with reference to WHO growth charts. Metabolome-wide association scans for rapid growth and overweight on over 4500 metabolic features were performed using multiple adjusted logistic mixed-effect models and controlling the false discovery rate (FDR) at 5%. In addition, we performed a look-up analysis of 43 pre-annotated metabolites, previously associated with birthweight or rapid growth.

**Results:**

In the Metabolome-Wide Association Study analysis, we identified three and eight metabolites associated with rapid growth and overweight, respectively, after FDR correction. Higher levels of cholestenone, a cholesterol derivative produced by microbial catabolism, were predictive of rapid growth (*p* = 1.6 × 10^−3^). Lower levels of the branched-chain amino acid (BCAA) valine (*p* = 8.6 × 10^−6^) were predictive of overweight in childhood. The area under the receiver operator curve for multivariate prediction models including these metabolites and traditional risk factors was 0.77 for rapid growth and 0.82 for overweight, compared with 0.69 and 0.69, respectively, for models using traditional risk factors alone. Among the 43 pre-annotated metabolites, seven and five metabolites were nominally associated (*P* < 0.05) with rapid growth and overweight, respectively. The BCAA leucine, remained associated (1.6 × 10^−3^) with overweight after FDR correction.

**Conclusion:**

The metabolites identified here may assist in the identification of children at risk of developing obesity and improve understanding of mechanisms involved in postnatal growth. Cholestenone and BCAAs are suggestive of a role of the gut microbiome and nutrient signalling respectively in child growth trajectories.

## Introduction

Childhood obesity has become a global epidemic in developed as well as in developing countries [1], with significant long-term consequences on both physical and psychological health, social and economic outcomes [2]. Behavioural dimensions such as diet and physical activity, and an ‘obesogenic environment’ that shapes those behaviours, have contributed to the spread of childhood obesity [3, 4]. In the last decades, there has been a growing interest in the idea that the early life environment can have lasting effects on the physiology and metabolism of the fetus and is associated with the early metabolic programming of human health [5–7]. Recent studies have revealed that several in utero exposures such as maternal socioeconomic status, clinical and environmental factors are associated with growth in infancy and with the subsequent development of childhood overweight or obesity [8–13]. The prenatal environment can affect fetus weight homeostasis and may result in a ‘thrifty phenotype’ that stores excess calories and predisposes children to weight gain [14]. Hence, a metabolic signature at birth may help elucidate the mechanisms involved in metabolic health later in life.

Metabolomics, the profiling of circulating small molecules, has been increasingly applied to investigate biological mechanisms associated with childhood obesity [15, 16]. However, few studies have investigated metabolic changes in cord blood that may predict subsequent infant growth and overweight and obesity [17]. Isganaitis, Rifas-Shiman et al. [18] analysed the metabolome in cord blood plasma from 26 cases and 26 controls differing in their postnatal weight trajectories using targeted mass spectrometry (MS) analysis of 415 metabolites, nested in an American cohort. There was a trend for lower levels of tryptophan metabolites in children that followed a rapid growth to obesity at 7 years trajectory. Sorrow, Maguire et al. [19] similarly applied a targeted MS analysis of 384 metabolites in cord blood of 25 obese and non-obese American children at 3–5 years. Children with obesity had elevated lipid species, acetaminophen metabolites and acylcarnitines compared with non‐obese children, although no multiple testing correction was applied. Hellmuth, Uhl et al. [20] applied a range of targeted LC-MS assays to assess 209 metabolites in cord blood of 700 German children in relation to birthweight, postnatal weight gain and BMI throughout adolescence. Although many metabolites were associated with weight at birth, no associations with postnatal measures survived multiple testing correction. Although initial studies have so far been based on small numbers of children or limited numbers of molecules, they reveal the potential of metabolic profiling in detecting biomarkers and pathways related to rapid growth in infancy as well as to overweight and obesity in early childhood. Identifying markers that are predictive of obesity onset may assist in the development of targeted intervention programmes for at-risk groups of children.

In this study, we have investigated the cord blood metabolic signatures of rapid growth in infancy and overweight in early childhood in four European birth cohorts, using untargeted LC-MS-based metabolic profiling. Our aims were twofold: firstly, to identify markers associated with rapid growth and overweight risk to provide mechanistic insight and elucidate causal pathways to obesity; and secondly to improve prediction of obesity risk in neonates through assessment of the predictive performance of models incorporating identified metabolites, in comparison with models based on traditional risk factors alone.

## Materials and methods

### Study population

The study population included participants from four population-based birth participating in the STOP project: ENVIRONAGE [21] (Belgium), INMA [22] (Spain), Piccolipiu [23] (Italy) and Rhea [24] (Greece). Ethical approval was obtained from the local Research Ethics Committees from each centre. Informed consent was obtained from the parents of the children. Further details of blood sampling, clinical, dietary and socioeconomic data of cohort individuals are given in the respective references and supporting information 1.

### Untargeted metabolomics

Cord blood samples were analysed in randomised order as a single uninterrupted batch with a UHPLC-QTOF-MS system (Agilent Technologies), as previously described [25]. Further details of the acquisition and structural annotation of features are given in supporting information 1.

### Outcome assessment

Rapid growth in infants in the first 12 months was categorised based on the definition of Ong et al. [25]. According to this definition, a clinically significant increment that indicates rapid growth occurs when there is a gain in weight of at least 0.67 standard deviations between different target ages. In this study, length data at birth were not available. Hence, rapid growth was defined as the weight *z* score change of >0.67 standard deviations (SD) between birth and twelve months of age based on World Health Organisation (WHO) growth charts [26]. A two-step prediction approach was used for calculating sex- and age-specific weight at exactly 12 months, using fractional polynomials of age by gender in each cohort [27] (supporting information 1).

To maintain sample size for the analysis of overweight in early childhood, we used a single measurement at an age greater than four years and as close to 6 years as available. The classification for healthy and overweight was based on WHO sex-adjusted and age-adjusted BMI *z* scores. WHO provides different classifications scheme for children under the age of 5 years (0–5< years) [28] and over the age of 5 years (5–18 years) [29]. Following the WHO proposed classification by De Onis and Lobstein [30], children younger than 5 years were classified as overweight if they had a BMI *z* scores >1 SD and children over 5 years were classified as overweight if they had a BMI z-score greater than 2 SDs [30].

### Statistical analysis

A Metabolome-Wide Association Study (MWAS) was applied to investigate the association between cord blood metabolomics and infant rapid growth/childhood overweight using multiple mixed-effect logistic regression models using the lme4 R package [31]. The basic model (Model 1) was adjusted for sex and age of the child at outcome measurement, ethnicity and we used a random-effect for cohort. To account for multiple testing, a Benjamini–Hochberg false discovery rate (FDR) [32] was applied using a cutoff of 5%.

We then applied additional covariate adjustment to significant features identified in the MWAS analysis. A directed acyclical graph was used to visualise assumptions regarding covariates for further model adjustment (Figure S1). Covariates were chosen based on a bivariate analysis of their correlation with outcomes (Logistic Regression). The resulting model (Model 2) included Model 1 covariates and maternal BMI, paternal BMI, gestational age, weight gained during pregnancy, paternal education, passive and active smoking status during pregnancy, parity and mode of delivery.

Pathway enrichment analysis on significant features was conducted using the *Mummichog* programme [33], supplemented with manual curation of the metabolite identities assigned by *Mummichog* (supporting information 1).

A look-up analysis, using the same statistical approach as the MWAS analysis (including 5% FDR), was conducted on 43 metabolites that had been previously annotated in the same data set as used in this study, due to their associations with birthweight [34, 35] or because they had previously been reported to predict a rapid growth leading to overweight in childhood trajectory, and could also be identified with high confidence through retention time and MS/MS matching in our data set [18].

In sensitivity analyses, we re-ran Model 2 for metabolites associated with rapid growth or overweight, stratified by cohort, sex and size for gestational age and additionally adjusted for birthweight.

We further assessed how well rapid growth in infancy or overweight in early childhood are predicted using metabolites in comparison with traditional factors using Random Forest classification models [36] (supporting information 1). We used three different sets of variables for each of the outcomes: (1) traditional risk factors (sex, birthweight, ethnicity, maternal BMI, paternal BMI, gestational age, maternal weight gain during pregnancy, paternal education, maternal passive and active smoking status during pregnancy, parity and mode of delivery), (2) significantly associated metabolites from the MWAS analysis and (3) significantly associated metabolites from MWAS analysis in combination with traditional risk factors. A bootstrap method of 1000 repetitions was advocated to quantify optimism and evaluate the generalisation of the model. A threefold cross-validation routine was performed on the training set (random 80% of the total observations) to each model to determine the optimum probability threshold. The model performance was evaluated on the relevant test set (remaining 20% of the total observations) using receiver operating characteristic (ROC curve) and area under the curve or AUROC for assessing the goodness-of-fit of the classifier. To further evaluate the predictive model, we performed a leave‐one‐out analysis by repeating the modelling process on a combined data set with one cohort retained as the validation set (supporting information 1).

## Results

### Participant Information and metabolomic data

Table 1 shows the characteristics of the population used in the analysis of rapid growth in infancy and overweight in early childhood (stratification by cohort, including available dietary information, is presented in Table S1, S2. In bivariate analyses (Table 1), birthweight, parity, maternal weight gained during pregnancy, mode of delivery and gestational age were all significantly associated (*P* < 0.05) with rapid growth, while maternal passive and active smoking during pregnancy, maternal BMI, paternal education level, paternal BMI and rapid growth in infancy were significantly associated with overweight in early childhood. After data filtering procedures, 4714 metabolic features were available for statistical analysis.

**Table 1:** Demographic, anthropometric and clinical outcome variables.

	Rapid growth at 12 months analysis			Overweight/obesity at early childhood analysis		
	(*n* = 391)	Missing	*p* value	(*n* = 272)	Missing	*p* value
*Cohort*						
RHEA	100 (25.6%)			97 (35.7%)		
ENVIRONAGE	109 (27.9%)			- ^b^		
Piccolipiu	95 (24.3%)			79 (29.0%)		
INMA	87 (22.3%)			96 (35.3%)		
*Gender*		0 (0%)	0.910		0 (0%)	0.801
Male	204 (52.2%)			145 (53.3%)		
Female	187 (47.8%)			127 (46.7%)		
*Birthweight (grams)*		0 (0%)	3.34E-15		0 (0%)	0.191
Mean (SD)	3295 (445)			3265 (412)		
*Maternal parity before this pregnancy*		3 (0.8%)			3 (1.1%)	
Nulliparous	182 (46.5%)		0.043	114 (41.9%)		0.113
Uniparous	169 (43.2%)		0.417	120 (44.1%)		0.170
Multiparous	37 (9.5%)			35 (12.9%)		
*Maternal active smoking*		1 (0.3%)	0.608		2 (0.7%)	0.045
No	324 (82.9%)			216 (79.4%)		
Yes	66 (16.8%)			54 (19.9%)		
*Maternal passive smoking*		8 (2.0%)	0.153		6 (2.2%)	7.10E-05
No	233 (59.6%)			123 (45.2%)		
Yes	150 (38.4%)			142 (52.2%)		
*Maternal BMI (kg/m*^*2*^*)*		1 (0.3%)	0.392		1 (0.4%)	7.93E-04
Mean (SD)	23.9 (4.58)			23.7 (4.51)		
*Maternal weight gain (kilograms)*		12 (3.1%)	0.021		11 (4.0%)	0.090
Mean (SD)	13.6 (5.16)			13.3 (5.09)		
*Delivery*		1 (0.3%)	0.040		1 (0.3%)	0.353
Vaginal	284 (72.6%)			170 (62.5%)		
Caesarean	106 (27.1%)			101 (37.1%)		
*Gestational age (weeks)*		0 (0%)	6.73E-11		0 (0%)	0.054
Mean (SD)	39.2 (1.61)			39.3 (1.56)		
*Mother born in cohort country*		0 (0%)	0.930		1 (0.3%)	0.483
No	35 (9.0%)			13 (4.8%)		
Yes	354 (90.5%)			259 (95.2%)		
*Father’s education*		13 (3.3%)			3 (1.1%)	
Primary school	70 (17.9%)		0.296	58 (21.3%)		0.202
Secondary school	189 (48.3%)		0.282	138 (50.7%)		0.019
University or higher	119 (30.4%)			73 (26.8%)		
*Paternal BMI (kg/m*^*2*^*)*		7 (1.8%)	0.139		3 (1.1%)	1.09E-04
Mean (SD)	25.8 (3.46)			26.0 (3.62)		
*Rapid growth*		0 (0%)			12 (4.4%)	1.13E-03
No	280 (71.1%)			168 (61.8%)		
Yes	114 (28.9.0%)			92 (33.8%)		
*Overweight/obesity in childhood*^*c*^		-			0 (0%)	
No	-			224 (82.4%)		
Yes				48 (17.6%)		
*Age at childhood BMI measurement (years)*	-	-			0 (0%)	
Mean (SD)	-	-		5.43 (1.00)		

Values are given in mean (standard deviation, SD) or percent (%).

^a^*p* value for association with rapid growth at 12 months of age and overweight in early childhood calculated from logistic regression.

^b^ENVIRONAGE was not included in the analysis of overweight in childhood as follow-up assessment was only available until 2 years.

^C^The classification for healthy and overweight was based on WHO sex-adjusted and age-adjusted BMI *z* scores.

### Cord blood metabolomics and rapid growth in the first year of life

**Fig. 1:**
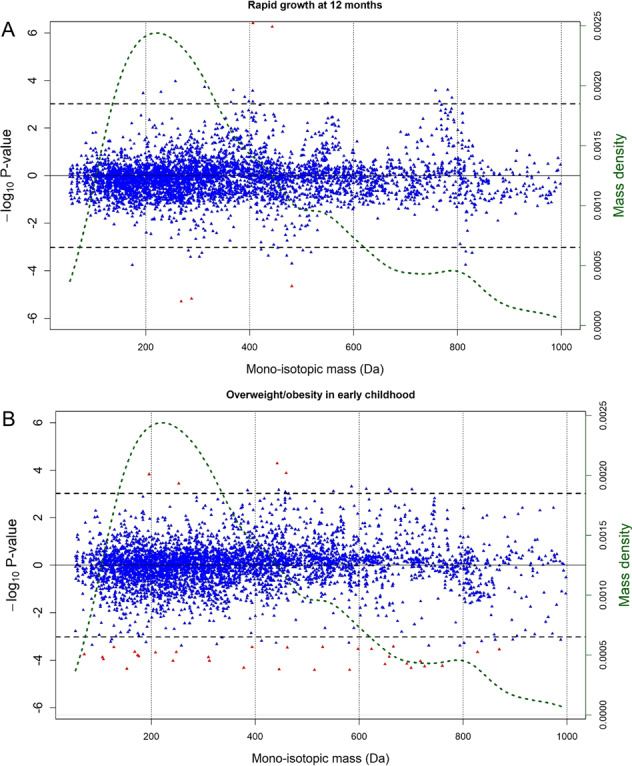
Metabolome wide associations with rapid growth and overweight. Signed Manhattan-type plot presenting the analysis of the 4714 UPLC-MS metabolic features for Model 1 for **A** rapid growth at twelve months of age and for **B** overweight in early childhood. The red dots represent the features that remain significant after applying the FDR threshold of 5%, whereas blue dots do not. The vertical axis shows the signed −log10 *P* value. The horizontal axis represents the monoisotopic mass (in Da). UPLC-MS-associated metabolic features are available in Table S3 and Table S6. The dotted green line represents the mass density.

**Fig. 2:**
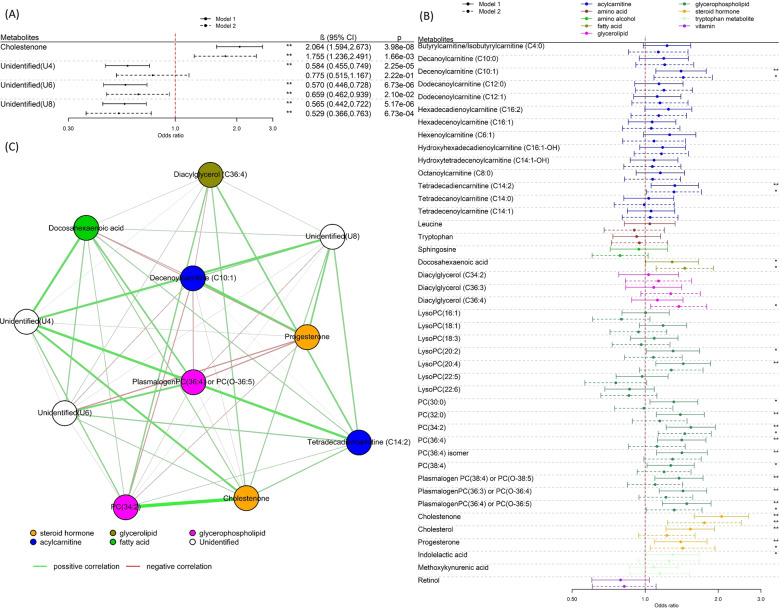
Metabolite associations with rapid growth. **A** Regression coefficients per standard deviation (95% confidence interval) between features and rapid growth at 12 months across all four cohorts (*N* = 391), identified in MWAS analysis. **B** Regression coefficients per standard deviation (95% confidence interval) between 43 pre-annotated metabolites and rapid growth at twelve months across all four cohorts (*N* = 391). The solids lines represent the results of Model 1 (adjusted for cohort and ethnicity) and the dotted lines the results of Model 2 (Model 1 further adjusted for maternal BMI, paternal BMI, gestational age, weight gained during pregnancy, paternal education passive and active smoking status during pregnancy, parity as well as a delivery mode). The * declares *P* < 0.05 while **FDR < 0.05. **C** Network graph (Pearson correlations) of metabolites associated with rapid growth at 12 months of age.

The analysis of rapid growth included 391 children, with 114 (28.9%) classed as rapid growers in the first year of life. In MWAS analysis, adjusting for age at the outcome measurement, sex, cohort and ethnicity (Model 1), six metabolic features were significantly associated (FDR < 5%) with rapid growth in the first year of life (Fig. 1A). Table S3 contains the retention time as well as the exact mass of all significantly associated features, including unassigned metabolites. The metabolic features were grouped into four metabolites after grouping of ions originating from the same molecule (matched by retention time and pairwise feature correlation, Table S3). One metabolite could be identified as cholestenone (4-cholesten-3-one; HMDB0000921), a steroid lipid in the class of cholesterols. Upon adjustment for further covariates (Model 2), three of the four associated metabolites, including cholestenone, remained significantly associated with rapid growth (Fig. 2A). Cholestenone levels were higher in the cord blood of rapid growers, whereas levels of the rest of the metabolites were lower in the cord blood of rapid growers.

In a look-up analysis, we analysed associations with 43 known metabolites (retention time and *m/z* information given in Table S4) in the metabolome data set that had been previously annotated based on their associations with birthweight [34, 35], or with rapid infancy weight gain and childhood obesity [18] (including indolelactic acid, sphingosine, tryptophan and leucine)(Table S5). Fourteen metabolites were associated with rapid growth in the first year of life (Fig. 2B) after correcting for 5% FDR in basic adjustment analyses (Model 1), including higher levels of nine phosphatidylcholines (PCs) or LysoPCs, cholestenone, cholesterol, progesterone and two acylcarnitines tetradecadiencarnitine (C14:2) and decenoylcarnitine (C10:1). In additionally adjusted analyses (Model 2) cholestenone, two PCs (PC(34:2) and plasmalogen PC(36:4)/PC(O-36:5)), two acylcarnitines, docosahexaenoic acid (DHA), diacylglycerol (C36:4) and progesterone were nominally associated (P < 0.05) with rapid growth (Fig. 2B). Directions of association with rapid growth were opposite to directions observed previously with birthweight [34]. Correcting Model 2 for 5% FDR, only cholestenone remained associated with rapid growth in the first year of life.

As shown in the network graph (Fig. 2C), cholestenone was highly correlated with PC(34:2), moderately correlated with unidentified metabolite U4 and had weaker, positive correlations with the other rapid growth-associated metabolites. We noted strong correlations between DHA and plasmalogen PC(36:4)/PC(O-36:5) as well as between tetradecadiencarnitine (C14:2) and PC(34:2).

*Mummichog* analysis indicated enrichment among rapid growers in the ‘C21-steroid hormone biosynthesis and metabolism’ and ‘Androgen and oestrogen biosynthesis and metabolism’ pathways, with weaker support for enrichment of the ‘Urea cycle/amino group metabolism’ pathway (supporting information 1 and 2, Table S10).

### Cord blood metabolomics and overweight in early childhood

**Fig. 3:**
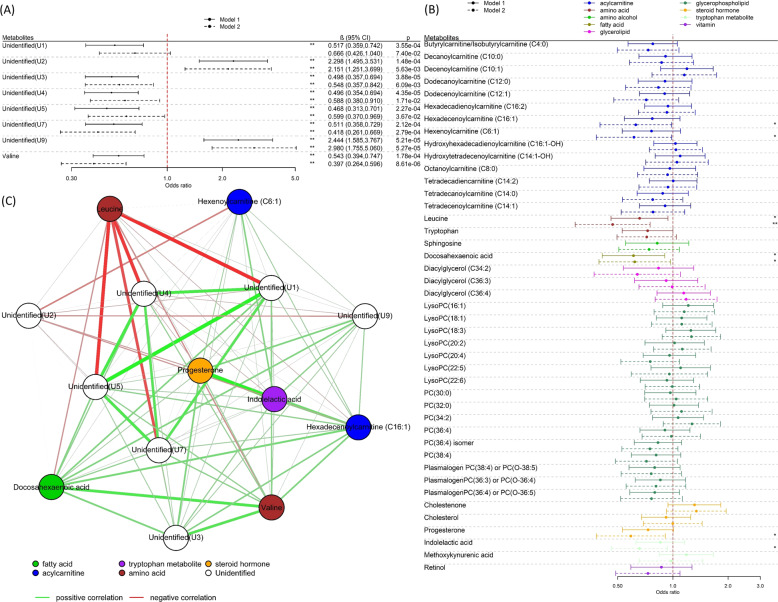
Metabolite associations with overweight. **A** Regression coefficients per standard deviation (95% confidence interval) between features with overweight in early childhood (*N* = 272), identified in MWAS analysis. **B** Regression coefficients per standard deviation (95% confidence interval) between 43 pre-annotated metabolites with overweight in early childhood (*N* = 272). The solids lines represent the results of Model 1 (adjusted for age of child at outcome measurement, cohort and ethnicity) and the dotted lines the results of Model 2 (Model 1 further adjusted for maternal BMI, paternal BMI, gestational age, weight gained during pregnancy, paternal education passive and active smoking status during pregnancy, parity as well as delivery mode). The * declares *P* < 0.05 while **FDR < 0.05. **C** Network graph (Pearson correlations) of metabolites associated in early childhood.

The analysis of child overweight in early childhood included 272 children from the Piccolipiu, Rhea and INMA cohorts, of which 48 (17.6%) were classed as being overweight or obese (mean age at weight status assessment: 5.12 years (SD:1.11)). In the MWAS, adjusting for cohort and ethnicity (Model 1), 36 features were significantly associated (FDR < 5%) with overweight in early childhood (Fig. 1B). After grouping ions originating from the same compound (Table S6), there were eight unique compounds associated with overweight (Fig. 3A). One compound could be annotated as valine, a branched-chain amino acid. Retention time as well as exact mass of all significantly associated features, including unassigned compounds, are available in Table S6. The inverse association of valine with overweight was strengthened upon additional covariate adjustment (Model 2) and remained significant after FDR correction.

In an analysis of the 43 pre-annotated metabolites, leucine and DHA were nominally associated (*P* < 0.05) with overweight in basic analyses (Model 1) (Fig. 2B). In additionally adjusted analyses (Model 2) lower levels of leucine, progesterone, indolelactic acid, hexenoylcarnitine (C6:1), hexadecenoylcarnitine (C16:1) and DHA were nominally associated (*P* < 0.05) with overweight in early childhood (Table S7). Directions of association with overweight were consistent with directions observed previously with birthweight [34]. Only leucine, a BCAA previously identified in relation to rapid infancy weight gain and childhood obesity by Isganaitis, Rifas-Shiman et al. [18], remained significant after FDR correction.

Valine was moderately correlated with DHA and had weaker correlations with the unidentified compounds U4, U5 and U7 and stronger correlations with U3 and hexadecenoylcarnitine (C16:1). Leucine had a weak negative correlation with Valine and strong negative correlations with U1, U4, U5 and U7. Strong correlations were observed between progesterone and indolelactic acid as well as between the compounds U1, U4, U5 and U7 (Fig. 3C).

*Mummichog* analysis did not provide strong support for enrichment of specific pathways with childhood overweight (supporting information 1 and 3, Table S11).

### Multivariate prediction models

**Fig. 4:**
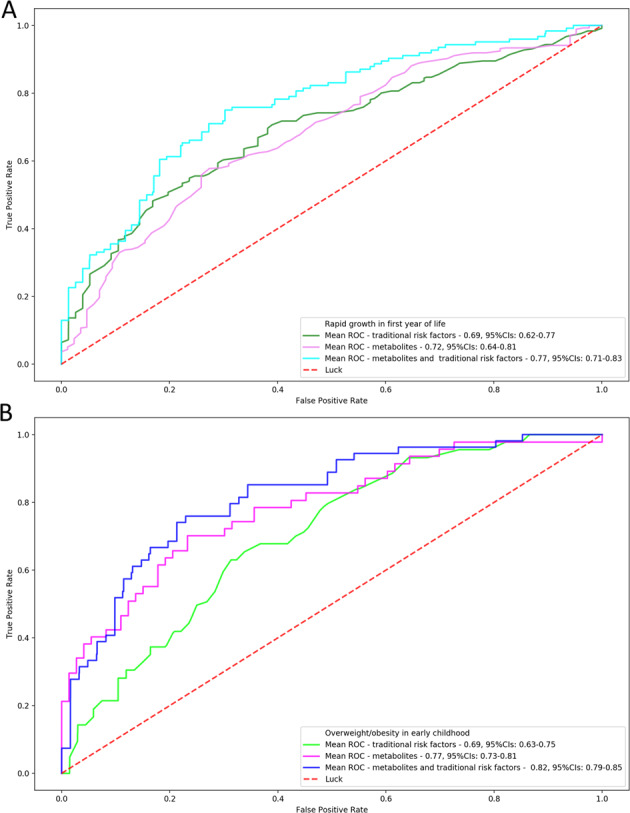
Multivariate prediction models of rapid growth and overweight. ROC mean value of 1000 bootstrapped model of threefolds for **A** rapid growth at 12 months of age after grouping the ions (nine metabolites) (population size: *N* = 391) and **B** overweight throughout early childhood (population size: *N* = 272).

We next utilised Random Forest classification models to evaluate the predictive performance of three different input variable sets for each of the two outcomes (Fig. 4). The rapid growth prediction model trained using only traditional risk factors exhibited a moderate predictive ability of an AUROC value of 0.69 (bootstrap 95% confidence interval (CI):0.62–0.77) (Table S8). Adding the four metabolites (cholestenone, U2, U4 and U8) identified in the MWAS analysis into the prediction model, increased the AUROC to 0.77 (bootstrap 95% CI: 0.73–0.81) (Fig. 4A). For overweight, using traditional risk factors alone, the AUROC was 0.69 (bootstrap 95%CI: 0.63–0.75), while a model using only the eight metabolites, Valine, U1, U2, U3, U4, U5, U7 and U9, identified in the MWAS analysis had an AUROC of 0.77 (bootstrap 95% CI: 0.73–0.81) (Table S8). The combined traditional risk factor and metabolite model was strongly predictive of overweight with an AUROC of 0.82 (bootstrap 95% CI: 0.79–0.85) (Fig. 4B). The leave cohort out analysis also showed improvement in predictive performance using metabolites, in the majority of cohorts (Table S9).

### Sensitivity analysis

To assess the robustness and consistency of our results, we stratified our population by cohort and by sex and repeated the adjusted models (Model 2) across each subpopulation. Regarding rapid growth, results were generally consistent across cohorts for all identified metabolites, including cholesterone (Figure S2). However, opposite directions of effects were observed in the Piccolipiu cohort for PC(34:2) and plasmalogen PC(36:4)/PC(O-36:5). Regarding overweight, results were again consistent across cohorts (Figure S3), although wide confidence intervals were observed in Piccolipiu (related to the small number of overweight cases available in this cohort). For valine, strong associations were noted in both the INMA and Rhea cohorts. For rapid growth, stronger associations were observed in boys with PC(34:2) and diacylglycerol (C36:4), while in girls stronger associations with rapid growth were observed with progesterone, tetradecadiencarnitine (C14:2), decenoylcarnitine(C10:1) and DHA (Figure S4). Very similar associations were seen with overweight upon stratification by sex (Figure S5).

To assess the role of birthweight in observed associations, we additionally adjusted our models for birthweight. There was some attenuation in effect size in associations for rapid growth (Figure S6), however, the attenuation with cholestenone was modest and significance was retained. Adjustment for birthweight had little effect on associations with overweight (Figure S7). Upon stratification by size for gestational age (< and ≥33rd percentile of birthweight for gestational age, Figure S8) we observed stronger associations with cholestenone and rapid growth as well as DHA and rapid growth among larger for gestational age (≥33rd percentile) infants. We noted stronger associations with hexadecenoylacarnitine (C16:1), hexenoylcarnitine(C6:1), leucine and valine and overweight among smaller for gestational age (<33rd percentile) infants (Figure S9).

## Discussion

This is the first study to date that investigates the association between untargeted metabolic profiles of cord blood and rapid growth at the first year of life and overweight/obesity in early childhood. We identified cholestenone and BCAA levels in cord blood as predictive of rapid growth and overweight/obesity, respectively, among healthy deliveries from four European populations. In multivariate analysis, we found that the addition of metabolites substantially improved prediction of both rapid growth and overweight compared with models using traditional risk factors alone.

Higher levels of cholestenone were identified as predictive of rapid growth in the MWAS analysis, with consistent effects noted across the four included cohorts. Little is known about the effects of cholestenone on health. It has previously been reported to be associated with CpG sites that are differentially methylated in relation to birthweight [35], however, birthweight did not appear to be an important contributor to the relationship between cholestenone and rapid growth in our study. Supplementation of diet with cholestenone leads to growth retardation in rodents and high levels cause hypertrophy of the adrenal glands, which may suggest potential endocrine effects [37, 38]. Cholestenone is produced by bacterial catabolism of cholesterol in the intestinal tract [39]. It therefore may be serving as a proxy indicator of the relative abundance of various microbiota present at birth, although the infant gut microbiome is generally uniform and under-developed at this stage [40]. Indeed, gestational age, which is known to influence the composition of the neonatal gut microbiome [41], was strongly associated with cholestenone levels in our data. However, the strong association between cholestenone and rapid growth remained after adjustment for gestational age. The role of the gut microflora in obesity is increasingly recognised [42] and differences in faecal microbiota composition measured during the first year of life have been found to be associated with weight status in later childhood [43].

Lower levels of the BCAAs valine and leucine were associated with overweight/obesity in early childhood, with consistent effects across both the Rhea and INMA cohorts. Associations were somewhat stronger with valine than leucine. Lower levels of cord blood leucine were also identified as nominally associated with children on a rapid growth trajectory by the study of Isganaitis, Rifas-Shiman et al. [18]. This is in contrast with the study of Hellmuth et al., where no associations were reported between BCAAs in cord blood and weight status at 2 and 10 years, although the authors speculated that the long storage period in their study may have degraded certain metabolites such as amino acids. BCAAs levels in cord blood represent the balance of supply, from the mother and from protein degradation, and of clearance through protein synthesis, excretion and BCAA catabolism and/or oxidation. BCAAs have a complex relationship with overweight and obesity. On one hand, higher levels in blood are consistently associated with obesity, insulin resistance and type 2 diabetes. Adjustment for maternal BMI, which would be expected to increase maternal levels and the fetal supply of BCAAs, strengthened the association between cord blood BCAA levels and childhood overweight, suggesting some negative confounding. On the other hand, numerous intervention studies and animal studies have shown that increasing dietary intake of BCAAs has beneficial signalling effects, with positive effects on parameters including body composition, glycemia and satiety [44]. Multiple mechanisms for these positive effects have been proposed including direct effects on hypothalamic and brainstem processes involved in satiety [44]. Cord blood BCAAs levels could therefore influence later propensity for overweight through causal processes such as control of food intake or alternatively serve as a marker of other metabolic processes that influence both propensity for weight gain and levels of BCAAs.

Apart from the association between leucine and overweight, no other associations were observed for metabolites identified by Isganaitis, Rifas-Shiman et al. [18]. Among metabolites previously identified as associated with birthweight, we identified higher levels of progesterone, PC(34:2), plasmalogen PC(36:4)/PC(O-36:5), DHA, decenoylcarnitine (C10:1), tetradecadiencarnitine (C14:2) and diacylglycerol (C36:4) as nominally associated with rapid growth, although these did not pass multiple testing correction. Progesterone is the major progestational hormone involved throughout all stages of pregnancy, and the pathway enrichment analysis also highlighted the role of hormonal signalling in rapid growth. DHA supplementation in milk has been shown to increase growth among preterm infants [45]. For overweight in early childhood, we noted nominal associations with lower levels of progesterone, indolelactic acid, hexenoylcarnitine (C6:1), hexadecenoylcarnitine (C16:1) and DHA. Indolelactic acid is a tryptophan catabolite that has an important role in the pathophysiology of obesity [46, 47] and is produced entirely by gut microbes [48]. Hexadecenoylcarnitine (C16:1) levels in the blood have been associated with obesity in children [49], while positive effects of DHA on obesity risk and metabolic health have been noted by multiple studies [50, 51], with proposed mechanisms including suppression of fatty-acid synthesis, enhancement of fatty-acid β-oxidation and increase of the serum adiponectin level [52]. The relatively small overlap in cord blood metabolites associated with birthweight and with rapid growth and with obesity, suggests that different mechanisms underlie these outcomes. Furthermore, despite the established association with rapid growth in infancy and later development of overweight, the different directions of effect in birthweight-related metabolites, observed with these two outcomes, suggest different contributory processes. Indeed, lower birthweight was a strong predictor of rapid growth while there was a trend for larger birthweight being associated with overweight in childhood.

Our analysis using a Random Forest classification model revealed that the coupling of the strongly associated molecules and demographic and clinical factors has a high ability to predict overweight/obesity in early childhood. Isganaitis, Rifas-Shiman et al. [18] suggested that cord blood metabolic signatures could be associated with early childhood obesity trajectories demonstrating, in a similar way with our analysis, that prediction models based on prenatal obesity factors (maternal age, pre-pregnancy BMI and breastfeeding duration) can be improved by adding cord blood associated metabolites. Although models would need to be validated in cohorts that are independent of the selection of metabolites, our results highlight a potential practical application of metabolomics to identify children at risk of obesity and support the potential merit of routine screening of cord blood [53].

A strength of our study includes the use of cord blood from multiple birth cohorts, enabling assessment of the metabolome prior to infant growth, limiting reverse causality. We included a number of prenatal sociodemographic and clinical factors in our analysis. However, we did not have complete data related to maternal nutrition and physical activity that could be linked to both the metabolome and the family environment later in life. Nevertheless, we used paternal socioeconomic factors and maternal clinical factors such as BMI that can reflect general patterns of family nutrition [54] and physical activity [55–57]. Future studies, with high-quality dietary data available, should explore the role of maternal nutrition on the cord blood metabolome.

Although the samples were analysed within a single analytical run in random order, we observed heterogeneity across the cohort metabolomic signatures, mainly explained by the processing of cord blood into plasma or serum. This heterogeneity can influence the observed associations, and for this reason, we added in the model a random effect variable for the cohort. Another limitation was that the sample was selected from the general population and we, therefore, had a relatively low number of overweight children. Furthermore, the use of BMI *z* scores to classify children as overweight is a blunter assessment of adiposity than direct measures such as dual-energy X-ray absorptiometry [58]. We used WHO obesity classification criteria, which have higher sensitivity and lower specificity in identifying obese subjects than the International Obesity Task Force cutoffs. The untargeted approach is both a strength and limitation: while it provides wide metabolome coverage [59], identification of the features can be challenging. Indeed, we were also unable to characterise all the significant features in the MWAS analysis.

## Conclusion

We have demonstrated metabolic profiles associated with rapid growth in infancy and overweight/obesity in early childhood, highlighting the role of multiple metabolites in various pathways. We presented evidence that cholestenone and BCAAs are associated with rapid growth in infancy and overweight/obesity in early childhood, respectively, and provide new insights on the potential mechanism underlying overweight risk, particularly early in development. Our findings present a potential route to the identification of at-risk children for the provision of targeted interventions to improve outcomes for children living in obesogenic environments.

## Supplementary information

Supporting information 1

Supporting information 2

Supporting information 3
