# Supplementary material for: Cord blood metabolic signatures predictive of childhood overweight and rapid growth
Source: Int J Obes (Lond). 2021 Jul 12;45(10):2252–60. doi: 10.1038/s41366-021-00888-1 (PMC8455328; doi:10.1038/s41366-021-00888-1)
Supplement: Supplementary file 1 — Supporting information 1 [file 41366_2021_888_MOESM1_ESM.docx]

Cord blood metabolic signatures predictive of childhood overweight and rapid growth

Evangelos Handakas^1^, Pekka Keski-Rahkonen^2^, Lida Chatzi^3^, Rossella Alfano^1,4^, Theano Roumeliotaki^5^, [Michelle Plusquin](javascript:;)^4^, Léa Maitre^6,7,8^, Lorenzo Richiardi^9^, Sonia Brescianini^10^, Augustin Scalbert^2^, Nivonirina Robinot^2^,Tim Nawrot^4^, Franco Sassi^11^, Martine Vrijheid^6,7,12^, Paolo Vineis^1^, Oliver Robinson^1,*^

^1^Μedical Research Council Centre for Environment and Health, School of Public Health, Imperial College London, London, United Kingdom

^2^Nutrition and Metabolism Section, International Agency for Research on Cancer, Lyon, France

^3^Department of Preventive Medicine, Keck School of Medicine, University of Southern California, Los Angeles, United States of America

^4^Centre for Environmental Sciences, Hasselt University, Diepenbeek, Belgium

^5^Department of Social Medicine, Faculty of Medicine, University of Crete, Heraklion, Greece

^6^Barcelona Institute of Global Health (ISGlobal), Barcelona, Spain

^7^Universitat Pompeu Fabra, Barcelona, Spain

^8^CIBER Epidemiología y Salud Pública (CIBERESP), Madrid, Spain

^9^Cancer Epidemiology Unit, Department of Medical Sciences, University of Turin and CPO‐Piemonte, Torino, Italy

^10^Centre for Behavioural Science and Mental Health, Istituto Superiore di Sanità, Rome, Italy

^11^Centre for Health Economics & Policy Innovation, Department of Economics & Public Policy, Imperial College Business School, South Kensington Campus, London, UK

^12^ Consorcio de Investigacion Biomedica en Red de Epidemiologia y Salud Publica (CIBERESP), Madrid, Spain

# Supporting information

Table of contents

[Supporting information 2](#_Toc72840014)

[1. Study population 20](#_Toc72840015)

[2. Untargeted metabolomics 22](#_Toc72840016)

[3. Random Forest and model evaluation for optimism 23](#_Toc72840017)

[4. Metabolic pathway enrichment analysis 26](#_Toc72840018)

[5. Modelling of weight and height growth trajectories 26](#_Toc72840019)

[References 31](#_Toc72840020)

List of figures and tables

[Table S1: Demographic, anthropometric and clinical outcome variables. 4](#_Toc72840101)

[Table S2: Individual number, observation number, demographic, anthropometric and clinical outcome variables average values (standard deviation) or percent (%) for overweight throughout early childhood by cohort. 5](#_Toc72840102)

[Table S3: All metabolomic features significantly associated (FDR 5%) with rapid growth at in first year of life. 6](#_Toc72840103)

[Table S4: Pre-annotated metabolites in cord blood that have been previously identified in the same dataset associated with birthweight (Robinson et al.; Alfano et al.), or because they have previously been reported to predict rapid growth leading to overweight in childhood trajectory (Isganaitis et al.). 7](#_Toc72840104)

[Table S5: Logistic regression odds ratio per standard deviation (95% CI) for rapid growth at twelve months for Model 1* and 2** for the birthweight related metabolites. 8](#_Toc72840105)

[Table S6: All metabolomic features significantly associated (FDR 5%) with overweight/obesity at early childhood. 9](#_Toc72840106)

[Table S7: Logistic regression odds ratio per standard deviation (95% CI) for overweight/obesity in early childhood for Model 1* and 2** for the birthweight related metabolites. 10](#_Toc72840107)

[Figure S1: Directed acyclical graph (DAG) to visualise assumptions regarding covariates, metabolome and outcome.. 11](#_Toc72840108)

[Figure S2: Logistic regression odds ratio per standard deviation (95% CI) for rapid growth for Model 2 stratified by cohort 12](#_Toc72840109)

[Figure S3: Logistic regression odds ratio per standard deviation (95% CI) for overweight/obesity in early childhood for Model 2 stratified by cohort 13](#_Toc72840110)

[Figure S4: Logistic regression odds ratio per standard deviation (95% CI) for rapid growth for Model 2 stratified by sex. 14](#_Toc72840111)

[Figure S5: Logistic regression odds ratio per standard deviation (95% CI) for overweight/obesity in early childhood for Model 2 stratified by sex 15](#_Toc72840112)

[Figure S6: Logistic regression odds ratio per standard deviation (95% CI) for rapid growth at twelve months for Model 1, Model 2, and Model 3 for the 7 nominal statistically significant birthweight related metabolites and the 4 associated with rapid growth at twelve months. 16](#_Toc72840113)

[Figure S7: Logistic regression odds ratio per standard deviation (95% CI) for overweight/obesity in early childhood for Model 1, Model 2 and Model 3 for the 6 nominal statistically significant birthweight related metabolites and the 8 associated with overweight/obesity in early childhood. 17](#_Toc72840114)

[Figure S8: Logistic regression odds ratio per standard deviation (95% CI) for rapid growth for Model 2 tratified by Small of Gestational Age (SGA) 18](#_Toc72840115)

[Figure S9: Logistic regression odds ratio per standard deviation (95% CI) for overweight/obesity in early childhood for Model 2 stratified by Small of Gestational Age (SGA) 19](#_Toc72840116)

[Table S8: Summary of rapid growth and 12 months of age and overweight/obesity in childhood. Average AUROC across 1000 bootstrapped test sets for all the cohorts. 24](#_Toc72840117)

[Table S9: Summary of rapid growth and 12 months of age and overweight/obesity in childhood. Average ROC and CI95% across 1000 bootstrapped test sets using and leave-cohort-out approach. 24](#_Toc72840118)

[Figure S10: PCA analysis of the whole metabolome and scatter plot of first two principal components, coloured by cohort. 25](#_Toc72840119)

[Table S10: Mummichog analysis statistically significant pathways for rapid growth at 12 months of age. 27](#_Toc72840120)

[Table S11: Mummichog analysis statistically significant pathways for overweight/obesity in early childhood. 28](#_Toc72840121)

[Table S12: Comparison of prediction concordance from different fractional polynomial powers for sex-specific weight and height in participating cohorts. 29](#_Toc72840122)

[Figure S11: Actual vs Predicted values of weight and height in participating ENVIRONAGE cohort. 30](#_Toc72840123)

[Figure S12: Actual vs Predicted values of weight and height in participating INMA cohort. 30](#_Toc72840124)

[Figure S13: Actual vs Predicted values of weight and height in participating Piccolipiu cohort. 30](#_Toc72840125)

[Figure S14: Actual vs Predicted values of weight and height in participating RHEA cohort. 31](#_Toc72840126)

Table S1: Demographic, anthropometric and clinical outcome variables. Values are given in mean (standard deviation, SD) or percent (%) for rapid growth at twelve months of age by cohort.

|  |  | |  | |  | |  | |
| --- | --- | --- | --- | --- | --- | --- | --- | --- |
|  | **RHEA (n=100)** | **Missing** | **ENVIRONAGE (n=109)** | **Missing** | **Piccolipiu (n=95)** | **Missing** | **INMA**  **(n=87)** | **Missing** |
| **cohort** |  |  |  |  |  |  |  |  |
| RHEA | 100 (100%) |  | - |  | - |  | - |  |
| ENVIRONAGE | - |  | 109 (100%) |  | - |  | - |  |
| Piccolipiu | - |  | - |  | 95 (100%) |  | - |  |
| INMA | - |  | - |  | - |  | 87 (100%) |  |
| **gender** |  |  |  |  |  |  |  |  |
| male | 53 (53.0%) |  | 55 (50.5%) |  | 54 (56.8%) |  | 42 (48.3%) |  |
| female | 47 (47.0%) |  | 54 (49.5%) |  | 41 (43.2%) |  | 45 (51.7%) |  |
| **maternal parity before this pregnancy** |  | 2 (2.0%) |  | 0 (0%) |  | 0 (0%) |  | 1 (1.1%) |
| nulliparous | 28 (28.0%) |  | 65 (59.6%) |  | 45 (47.4%) |  | 44 (50.6%) |  |
| uniparous | 47 (47.0%) |  | 44 (40.4%) |  | 42 (44.2%) |  | 36 (41.4%) |  |
| multiparous | 23 (23.0%) |  | 0 (0%) |  | 8 (8.4%) |  | 6 (6.9%) |  |
| **maternal age** |  | 0 (0%) |  | 0 (0%) |  | 1 (1.1%) |  | 0 (0%) |
| Mean (SD) | 30.0 (4.99) |  | 29.1 (3.63) |  | 33.3 (4.46) |  | 31.7 (4.03) |  |
| **mother’s education** |  | 1 (1.0%) |  | 4 (3.7%) |  | 0 (0%) |  | 0 (0%) |
| primary school | 8 (8.0%) |  | 9 (8.3%) |  | 6 (6.3%) |  | 18 (20.7%) |  |
| secondary school | 57 (57.0%) |  | 29 (26.6%) |  | 40 (42.1%) |  | 40 (46.0%) |  |
| university or higher | 34 (34.0%) |  | 67 (61.5%) |  | 49 (51.6%) |  | 29 (33.3%) |  |
| **father’s education** |  | 2 (2.0%) |  | 10 (9.2%) |  | 0 (0%) |  | 1 (1.1%) |
| primary school | 21 (21.0%) |  | 10 (9.2%) |  | 16 (16.8%) |  | 23 (26.4%) |  |
| secondary school | 57 (57.0%) |  | 45 (41.3%) |  | 43 (45.3%) |  | 44 (50.6%) |  |
| university or higher | 20 (20.0%) |  | 44 (40.4%) |  | 36 (37.9%) |  | 19 (21.8%) |  |
| **maternal smoking** |  | 1 (1.0%) |  | 0 (0%) |  | 0 (0%) |  | 0 (0%) |
| no | 79 (79.0%) |  | 102 (93.6%) |  | 76 (80.0%) |  | 67 (77.0%) |  |
| yes | 20 (20.0%) |  | 7 (6.4%) |  | 19 (20.0%) |  | 20 (23.0%) |  |
| **passive smoke exposure** |  | 5 (5.0%) |  | 2 (1.8%) |  | 0 (0%) |  | 1 (1.1%) |
| no | 13 (13.0%) |  | 100 (91.7%) |  | 74 (77.9%) |  | 46 (52.9%) |  |
| yes | 82 (82.0%) |  | 7 (6.4%) |  | 21 (22.1%) |  | 40 (46.0%) |  |
| **maternal height (cm)** |  | 1 (1.0%) |  | 5 (4.6%) |  | 0 (0%) |  | 1 (1.1%) |
| Mean (SD) | 163 (5.65) |  | 167 (7.07) |  | 164 (5.67) |  | 163 (6.66) |  |
| **maternal weight (kg)** |  | 1 (1.0%) |  | 0 (0%) |  | 0 (0%) |  | 0 (0%) |
| Mean (SD) | 66.8 (15.6) |  | 67.4 (14.0) |  | 61.1 (11.2) |  | 63.1 (11.5) |  |
| **maternal BMI** |  | 1 (1.0%) |  | 0 (0%) |  | 0 (0%) |  | 0 (0%) |
| Mean (SD) | 25.1 (5.37) |  | 24.1 (4.52) |  | 22.7 (3.91) |  | 23.7 (3.99) |  |
| **maternal weight gain (kg)** |  | 11 (11.0%) |  | 0 (0%) |  | 1 (1.1%) |  | 0 (0%) |
| Mean (SD) | 13.1 (5.89) |  | 14.4 (5.20) |  | 12.4 (4.44) |  | 14.2 (4.82) |  |
| **paternal height (cm)** |  | 1 (1.0%) |  | 5 (4.6%) |  | 0 (0%) |  | 1 (1.1%) |
| Mean (SD) | 176 (7.21) |  | 179 (7.51) |  | 177 (6.31) |  | 177 (6.48) |  |
| **paternal weight (kg)** |  | 0 (0%) |  | 5 (4.6%) |  | 0 (0%) |  | 1 (1.1%) |
| Mean (SD) | 85.0 (14.5) |  | 81.2 (10.8) |  | 78.3 (9.65) |  | 81.3 (13.7) |  |
| **paternal age (years)** |  | 0 (0%) |  | 4 (3.7%) |  | 1 (1.1%) |  | 0 (0%) |
| Mean (SD) | 34.2 (5.04) |  | 31.7 (4.77) |  | 36.8 (5.48) |  | 33.6 (4.06) |  |
| **delivery** |  | 0 (0%) |  | 0 (0%) |  | 0 (0%) |  | 1 (1.1%) |
| vaginal | 38 (38.0%) |  | 103 (94.5%) |  | 64 (67.4%) |  | 79 (90.8%) |  |
| caesarean | 62 (62.0%) |  | 6 (5.5%) |  | 31 (32.6%) |  | 7 (8.0%) |  |
| **pregnancy diabetes** |  | 0 (0%) |  | 0 (0%) |  | 0 (0%) |  | 33 (37.9%) |
| no | 89 (89.0%) |  | 107 (98.2%) |  | 88 (92.6%) |  | 49 (56.3%) |  |
| yes | 11 (11.0%) |  | 7 (7.4%) |  | 11 (11.0%) |  | 2 (1.8%) |  |
| **birth weight (g)** |  | 0 (0%) |  | 0 (0%) |  | 0 (0%) |  | 0 (0%) |
| Mean (SD) | 3270 (428) |  | 3420 (551) |  | 3230 (406) |  | 3290 (402) |  |
| **gestational age (weeks)** |  | 0 (0%) |  | 0 (0%) |  | 0 (0%) |  | 0 (0%) |
| Mean (SD) | 38.4 (1.32) |  | 39.0 (1.61) |  | 39.6 (1.60) |  | 39.9 (1.54) |  |
| **ethnicity** |  | 0 (0%) |  | 0 (0%) |  | 0 (0%) |  | 0 (0%) |
| non native | 5 (5.0%) |  | 18 (16.5%) |  | 8 (8.4%) |  | 4 (4.6%) |  |
| native | 95 (95.0%) |  | 89 (81.7%) |  | 87 (91.6%) |  | 83 (95.4%) |  |
| **paternal BMI** |  | 1 (1.0%) |  | 5 (4.6%) |  | 0 (0%) |  | 1 (1.1%) |
| Mean (SD) | 27.2 (3.90) |  | 25.3 (3.10) |  | 24.9 (2.70) |  | 25.8 (3.63) |  |
| **breast feeding** |  | 4 (4.0%) |  | 109 (100%) |  | 3 (3.2%) |  | 0 (0%) |
| no | 12 (12.0%) |  | 0 (0%) |  | 12 (12.6%) |  | 7 (8.0%) |  |
| yes | 84 (84.0%) |  | 0 (0%) |  | 80 (84.2%) |  | 80 (92.0%) |  |
| **breast feeding duration (weeks)** |  | 4 (4.0%) |  | 109 (100%) |  | 19 (20.0%) |  | 0 (0%) |
| Mean (SD) | 19.3 (20.7) |  | NA (NA) |  | 42.5 (28.9) |  | 23.3 (17.4) |  |
| **rapid growth** |  | 0 (0%) |  | 13 (4.7%) |  | 0 (0%) |  | 0 (0%) |
| no | 60 (60.0%) |  | 76 (69.7%) |  | 82 (83.7%) |  | 62 (71.3%) |  |
| yes | 40 (40.0%) |  | 33 (30.3%) |  | 16 (16.3%) |  | 25 (28.7%) |  |
| **Vegetables (serves/day)** |  | 0 (0%) |  | 0 (0%) |  | 0 (0%) |  | 0 (0%) |
| Mean (SD) | 4.04 (2.87) |  | 1.79 (0.829) |  | 1.13 (0.630) |  | 2.36 (1.17) |  |
| **Fruits (serves/day)** |  | 0 (0%) |  | 109 (100%) |  | 0 (0%) |  | 0 (0%) |
| Mean (SD) | 2.12 (2.35) |  | NA (NA) |  | 0.956 (0.441) |  | 2.88 (1.58) |  |
| **Milk** **products (serves/day)** |  | 0 (0%) |  | 109 (100%) |  | 0 (0%) |  | 0 (0%) |
| Mean (SD) | 2.42 (1.45) |  | NA (NA) |  | 1.08 (0.593) |  | 3.08 (1.34) |  |
| **Fish (serves/day)** |  | 0 (0%) |  | 0 (0%) |  | 0 (0%) |  | 0 (0%) |
| Mean (SD) | 0.196 (0.198) |  | 2.21 (1.05) |  | 0.175 (0.101) |  | 0.774 (0.559) |  |
| **Pulses (serves/day)** |  | 0 (0%) |  | 109 (100%) |  | 0 (0%) |  | 0 (0%) |
| Mean (SD) | 0.422 (0.513) |  | NA (NA) |  | 0.198 (0.195) |  | 0.250 (0.286) |  |
| **Sugar (serves/day)** |  | 0 (0%) |  | 109 (100%) |  | 0 (0%) |  | 0 (0%) |
| Mean (SD) | 1.29 (1.33) |  | NA (NA) |  | 0.526 (0.378) |  | 4.04 (2.56) |  |
| **Eggs (serves/day)** |  | 0 (0%) |  | 109 (100%) |  | 0 (0%) |  | 0 (0%) |
| Mean (SD) | 0.178 (0.297) |  | NA (NA) |  | 0.179 (0.132) |  | 0.379 (0.179) |  |
| **Grains (serves/day)** |  | 0 (0%) |  | 109 (100%) |  | 0 (0%) |  | 0 (0%) |
| Mean (SD) | 3.15 (3.45) |  | NA (NA) |  | 1.17 (0.354) |  | 2.28 (0.974) |  |
| **Meat (serves/day)** |  | 0 (0%) |  | 109 (100%) |  | 0 (0%) |  | 0 (0%) |
| Mean (SD) | 0.477 (0.436) |  | NA (NA) |  | 1.30 (0.774) |  | 0.882 (0.344) |  |
| **Processed meat (serves/day)** |  | 10 (10.0%) |  | 109 (100%) |  | 0 (0%) |  | 0 (0%) |
| Mean (SD) | 0.340 (0.433) |  | NA (NA) |  | 0.222 (0.237) |  | 0.359 (0.303) |  |
| **Potatoes (serves/day)** |  | 0 (0%) |  | 109 (100%) |  | 0 (0%) |  | 0 (0%) |
| Mean (SD) | 0.596 (0.612) |  | NA (NA) |  | 0.228 (0.154) |  | 0.526 (0.317) |  |

Table S2: Individual number, observation number, demographic, anthropometric and clinical outcome variables average values (standard deviation) or percent (%) for overweight throughout early childhood by cohort.

|  |  | |  | | |  | | |  |
| --- | --- | --- | --- | --- | --- | --- | --- | --- | --- |
|  | **RHEA (n=97)** | **Missing** | | **Piccolipiu (n=79)** | **Missing** | | **INMA (n=96)** | **Missing** | |
| **cohort** |  |  | |  |  | |  |  | |
| RHEA | 97 (100%) |  | | - |  | | - |  | |
| ENVIRONAGE | - |  | | - |  | | - |  | |
| Piccolipiu | - |  | | 79 (100%) |  | | - |  | |
| INMA | - |  | | - |  | | 96 (100%) |  | |
| **age** **at weight status assessment** |  | 0 (0%) | |  | 0 (0%) | |  | 0 (0%) | |
|  | 5.53 (1.03) |  | | 4.43 (0.105) |  | | 6.16 (0.635) |  | |
|  | 6.02 [4.01, 7.07] |  | | 4.42 [4.17, 4.75] |  | | 6.14 [4.04, 7.49] |  | |
| **gender** |  |  | |  |  | |  |  | |
| male | 53 (54.6%) |  | | 44 (55.7%) |  | | 48 (50.0%) |  | |
| female | 44 (45.4%) |  | | 35 (44.3%) |  | | 48 (50.0%) |  | |
| **maternal parity before this pregnancy** |  | 2 (2.1%) | |  | 0 (0%) | |  | 1 (1.0%) | |
| nulliparous | 26 (26.8%) |  | | 37 (46.8%) |  | | 51 (53.1%) |  | |
| uniparous | 46 (47.4%) |  | | 37 (46.8%) |  | | 37 (38.5%) |  | |
| multiparous | 23 (23.7%) |  | | 5 (6.3%) |  | | 7 (7.3%) |  | |
| **maternal age (years)** |  | 0 (0%) | |  | 1 (1.3%) | |  | 0 (0%) | |
| Mean (SD) | 30.2 (4.94) |  | | 33.7 (4.61) |  | | 31.6 (4.09) |  | |
| Median [Min, Max] | 29.8 [20.3, 41.7] |  | | 33.9 [19.9, 42.8] |  | | 31.8 [23.6, 41.3] |  | |
| **mother’s education** |  | 1 (1.0%) | |  | 0 (0%) | |  | 0 (0%) | |
| primary school | 7 (7.2%) |  | | 4 (5.1%) |  | | 18 (18.8%) |  | |
| secondary school | 55 (56.7%) |  | | 33 (41.8%) |  | | 44 (45.8%) |  | |
| university or higher | 34 (35.1%) |  | | 42 (53.2%) |  | | 34 (35.4%) |  | |
| **father’s education** |  | 2 (2.1%) | |  | 0 (0%) | |  | 1 (1.0%) | |
| primary school | 20 (20.6%) |  | | 11 (13.9%) |  | | 27 (28.1%) |  | |
| secondary school | 55 (56.7%) |  | | 37 (46.8%) |  | | 46 (47.9%) |  | |
| university or higher | 20 (20.6%) |  | | 31 (39.2%) |  | | 22 (22.9%) |  | |
| **maternal smoking** |  | 1 (1.0%) | |  | 0 (0%) | |  | 1 (1.0%) | |
| no | 77 (79.4%) |  | | 65 (82.3%) |  | | 74 (77.1%) |  | |
| yes | 19 (19.6%) |  | | 14 (17.7%) |  | | 21 (21.9%) |  | |
| **passive smoke exposure** |  | 5 (5.2%) | |  | 0 (0%) | |  | 2 (2.1%) | |
| no | 13 (13.4%) |  | | 62 (78.5%) |  | | 48 (50.0%) |  | |
| yes | 79 (81.4%) |  | | 17 (21.5%) |  | | 46 (47.9%) |  | |
| **maternal height (cm)** |  | 1 (1.0%) | |  | 0 (0%) | |  | 2 (2.1%) | |
| Mean (SD) | 163 (5.56) |  | | 164 (6.04) |  | | 163 (6.59) |  | |
| **maternal weight (kg)** |  | 1 (1.0%) | |  | 0 (0%) | |  | 0 (0%) | |
| Mean (SD) | 67.1 (15.7) |  | | 59.8 (10.6) |  | | 62.3 (10.5) |  | |
| **maternal BMI** |  | 1 (1.0%) | |  | 0 (0%) | |  | 0 (0%) | |
| Mean (SD) | 25.2 (5.42) |  | | 22.2 (3.67) |  | | 23.4 (3.64) |  | |
| **maternal weight gain (kg)** |  | 11 (11.3%) | |  | 0 (0%) | |  | 0 (0%) | |
| Mean (SD) | 13.0 (5.78) |  | | 12.3 (4.19) |  | | 14.3 (4.97) |  | |
| **paternal height (cm)** |  | 1 (1.0%) | |  | 0 (0%) | |  | 2 (2.1%) | |
| Mean (SD) | 177 (7.14) |  | | 178 (6.24) |  | | 177 (6.80) |  | |
| **paternal weight (kg)** |  | 0 (0%) | |  | 0 (0%) | |  | 2 (2.1%) | |
| Mean (SD) | 85.1 (14.4) |  | | 79.1 (11.1) |  | | 81.1 (13.3) |  | |
| **paternal age (years)** |  | 0 (0%) | |  | 1 (1.3%) | |  | 0 (0%) | |
| Mean (SD) | 34.2 (5.02) |  | | 36.7 (5.50) |  | | 33.5 (4.32) |  | |
| **delivery** |  | 0 (0%) | |  | 0 (0%) | |  | 1 (1.0%) | |
| vaginal | 36 (37.1%) |  | | 50 (63.3%) |  | | 84 (87.5%) |  | |
| caesarean | 61 (62.9%) |  | | 29 (36.7%) |  | | 11 (11.5%) |  | |
| **pregnancy diabetes** |  | 0 (0%) | |  | 0 (0%) | |  | 41 (42.7%) | |
| no | 87 (89.7%) |  | | 73 (92.4%) |  | | 50 (52.1%) |  | |
| yes | 10 (10.3%) |  | | 6 (7.6%) |  | | 5 (5.2%) |  | |
| **birth weight (g)** |  | 0 (0%) | |  | 0 (0%) | |  | 0 (0%) | |
| Mean (SD) | 3270 (428) |  | | 3230 (406) |  | | 3290 (402) |  | |
| **gestational age (weeks)** |  | 0 (0%) | |  | 0 (0%) | |  | 0 (0%) | |
| Mean (SD) | 38.4 (1.30) |  | | 39.7 (1.49) |  | | 39.8 (1.51) |  | |
| Median [Min, Max] | 38.4 [34.2, 41.1] |  | | 39.6 [36.6, 44.6] |  | | 39.9 [34.3, 44.7] |  | |
| **ethnicity** |  | 0 (0%) | |  | 0 (0%) | |  | 0 (0%) | |
| native | 4 (4.1%) |  | | 5 (6.3%) |  | | 4 (4.2%) |  | |
| non native | 93 (95.9%) |  | | 74 (93.7%) |  | | 92 (95.8%) |  | |
| **paternal BMI** |  | 1 (1.0%) | |  | 0 (0%) | |  | 2 (2.1%) | |
| Mean (SD) | 27.2 (3.82) |  | | 24.9 (3.18) |  | | 25.8 (3.46) |  | |
| **breast feeding** |  | 4 (4.1%) | |  | 1 (1.3%) | |  | 0 (0%) | |
| no | 12 (12.4%) |  | | 10 (12.7%) |  | | 8 (8.3%) |  | |
| yes | 81 (83.5%) |  | | 68 (86.1%) |  | | 88 (91.7%) |  | |
| **breast feeding duration (weeks)** |  | 4 (4.1%) | |  | 16 (20.3%) | |  | 0 (0%) | |
| Mean (SD) | 18.8 (20.4) |  | | 42.9 (28.8) |  | | 23.1 (17.4) |  | |
| **overweight/obesity^a^ population** |  | 0 (0%) | |  | 0 (0%) | |  | 0 (0%) | |
| no | 66 (68.0%) |  | | 75 (94.9%) |  | | 67 (69.8%) |  | |
| yes | 31 (32.0%) |  | | 4 (5.1%) |  | | 29 (30.2%) |  | |
| **rapid growth** |  | 0 (0%) | |  | 0 (0%) | |  | 12 (12.5%) | |
| no | 59 (60.8%) |  | | 82 (83.7%) |  | | 60 (62.5%) |  | |
| yes | 38 (39.2%) |  | | 16 (16.3%) |  | | 24 (25.0%) |  | |
| **Vegetables (serves/day)** |  | 0 (0%) | |  | 0 (0%) | |  | 1 (1.0%) | |
| Mean (SD) | 4.09 (2.88) |  | | 1.13 (0.665) |  | | 2.31 (1.18) |  | |
| **Fruits (serves/day)** |  | 0 (0%) | |  | 0 (0%) | |  | 1 (1.0%) | |
| Mean (SD) | 2.15 (2.38) |  | | 0.951 (0.458) |  | | 2.81 (1.54) |  | |
| **Milk products (serves/day)** |  | 0 (0%) | |  | 0 (0%) | |  | 1 (1.0%) | |
| Mean (SD) | 2.42 (1.47) | 1.10 (0.574) | | 1.10 (0.574) |  | | 3.12 (1.30) |  | |
| **Fish (serves/day)** |  | 0 (0%) | |  | 0 (0%) | |  | 1 (1.0%) | |
| Mean (SD) | 0.195 (0.200) | 0.175 (0.106) | | 0.175 (0.106) |  | | 0.764 (0.551) |  | |
| **Pulses (serves/day)** |  | 0 (0%) | |  | 0 (0%) | |  |  | |
| Mean (SD) | 0.427 (0.519) |  | | 0.186 (0.157) |  | | 0.249 (0.277) | 1 (1.0%) | |
| **Sugar (serves/day)** |  | 0 (0%) | |  | 0 (0%) | |  |  | |
| Mean (SD) | 1.30 (1.34) |  | | 0.548 (0.389) |  | | 3.97 (2.54) |  | |
| **Eggs (serves/day)** |  | 0 (0%) | |  | 0 (0%) | |  | 1 (1.0%) | |
| Mean (SD) | 0.177 (0.298) |  | | 0.175 (0.115) |  | | 0.381 (0.173) |  | |
| **Grains (serves/day)** |  | 0 (0%) | |  | 0 (0%) | |  |  | |
| Mean (SD) | 3.12 (3.47) |  | | 1.18 (0.343) |  | | 2.23 (0.958) | 1 (1.0%) | |
| **Meat (serves/day)** |  | 0 (0%) | |  | 0 (0%) | |  |  | |
| Mean (SD) | 0.480 (0.442) |  | | 1.28 (0.774) |  | | 0.869 (0.336) |  | |
| **Processed meat (serves/day)** |  | 0 (0%) | |  | 0 (0%) | |  | 1 (1.0%) | |
| Mean (SD) | 0.337 (0.438) |  | | 0.222 (0.229) |  | | 0.344 (0.212) |  | |
| **Potatoes (serves/day)** |  | 0 (0%) | |  | 0 (0%) | |  | 1 (1.0%) | |
| Mean (SD) | 0.597 (0.617) |  | | 0.216 (0.143) |  | | 0.524 (0.313) |  | |

*^a^*Classification based on WHO sex-adjusted and age-adjusted BMI z-scores

Table S3: All metabolomic features significantly associated (FDR 5%) with rapid growth at in first year of life. In case of more than one feature per compound were detected, the feature with highest intensity is written in bold.

| **Compound** | ***m/z*** | **Rt(min)** | **Annotation** | **Estimate** | **Std Error** | **t-score** | **p-value*** |
| --- | --- | --- | --- | --- | --- | --- | --- |
| **1** | **385.3487** | **9.076708** | **Cholestenone** | **0.725** | **0.132** | **5.492** | **1.88E-04** |
| 1 | 407.3299 | 9.073516 | Cholestenone | 0.642 | 0.127 | 5.076 | 8.66E-04 |
|  |  |  |  |  |  |  |  |
| 2 | 269.1894 | 5.3084226 | Unidentified(U8) | -0.571 | 0.125 | -4.558 | 6.09E-03 |
|  |  |  |  |  |  |  |  |
| 3 | 289.2157 | 4.8316393 | Unidentified (U6) | -0.563 | 0.125 | -4.502 | 6.34E-03 |
|  |  |  |  |  |  |  |  |
| 4 | 482.2392 | 3.6582649 | Unidentified (U4) | -0.538 | 0.127 | -4.238 | 1.77E-02 |

*Model was adjusted for child’s sex and age at outcome measurement and ethnicity. We used a random *effects* model by cohort

Table S4: Pre-annotated metabolites in cord blood that have been previously identified in the same dataset associated with birthweight (Robinson et al.; Alfano et al.), or because they have previously been reported to predict rapid growth leading to overweight in childhood trajectory (Isganaitis et al.).

| **ID** | **Metabolite name** | ***m/z*** | **retention time (minutes)** | **Reference** |
| --- | --- | --- | --- | --- |
| **1** | Leucine | 132.1021 | 1.4519173 | Isganaitis et al., 2015 |
| **2** | Tryptophan | 205.0965 | 2.4842238 | Isganaitis et al., 2015 |
| **3** | Indolelactic acid | 206.0822 | 3.8289883 | Robinson et al., 2018 |
| **4** | Methoxykynurenic acid | 220.5393 | 3.6709497 | Robinson et al., 2018 |
| **5** | Butyrylcarnitine/Isobutyrylcarnitine (C4:0) | 232.1537 | 1.9274178 | Robinson et al., 2018 |
| **6** | Hexenoylcarnitine (C6:1) | 258.1699 | 2.8306587 | Robinson et al., 2018 |
| **7** | Retinol | 269.2278 | 7.2190323 | Robinson et al., 2018 |
| **8** | Octanoylcarnitine (C8:0) | 288.2171 | 4.4222255 | Robinson et al., 2018 |
| **9** | Sphingosine | 300.2905 | 6.4203 | Isganaitis et al., 2015 |
| **10** | Decenoylcarnitine (C10:1) | 314.2321 | 4.8776007 | Robinson et al., 2018 |
| **11** | Progesterone | 315.232 | 6.3944817 | Robinson et al., 2018 |
| **12** | Decanoylcarnitine (C10:0) | 316.2489 | 5.1387444 | Robinson et al., 2018 |
| **13** | Docosahexaenoic acid | 329.2482 | 7.2322 | Robinson et al., 2018 |
| **14** | Dodecenoylcarnitine (C12:1) | 342.2641 | 5.422301 | Robinson et al., 2018 |
| **15** | Dodecanoylcarnitine (C12:0) | 344.2797 | 5.647444 | Robinson et al., 2018 |
| **16** | Tetradecadiencarnitine (C14:2) | 368.2793 | 5.631012 | Robinson et al., 2018 |
| **17** | Cholesterol | 369.3521 | 9.60744 | Alfano et al., 2019 |
| **18** | Tetradecenoylcarnitine (C14:1) | 370.2955 | 5.840157 | Robinson et al., 2018 |
| **19** | Tetradecanoylcarnitine (C14:0) | 372.3112 | 6.56033 | Robinson et al., 2018 |
| **20** | Cholestenone | 385.3487 | 9.76708 | Alfano et al., 2019 |
| **21** | Hydroxytetradecenoylcarnitine (C14:1-OH) | 386.2899 | 5.568466 | Robinson et al., 2018 |
| **22** | Hexadecadienoylcarnitine (C16:2) | 396.31 | 5.9437513 | Robinson et al., 2018 |
| **23** | Hexadecenoylcarnitine (C16:1) | 398.3264 | 6.1093335 | Robinson et al., 2018 |
| **24** | Hydroxyhexadecadienoylcarnitine (C16:1-OH) | 412.3045 | 5.749766 | Robinson et al., 2018 |
| **25** | LysoPC(16:1) | 494.325 | 6.817081 | Robinson et al., 2018 |
| **26** | LysoPC(18:3) | 518.3216 | 6.7819257 | Robinson et al., 2018 |
| **27** | LysoPC(18:1) | 522.3555 | 6.979925 | Robinson et al., 2018 |
| **28** | LysoPC(20:2) | 548.3681 | 7.141414 | Robinson et al., 2018 |
| **29** | LysoPC(20:4) | 563.3141 | 6.8930106 | (Alfano et al., 2019; Robinson et al., 2018), |
| **30** | LysoPC(22:6) | 568.3409 | 6.88448 | Robinson et al., 2018 |
| **31** | LysoPC(22:5) | 570.3551 | 7.206504 | Robinson et al., 2018 |
| **32** | Diacylglycerol (C34:2) | 615.4959 | 9.762555 | Robinson et al., 2018 |
| **33** | Diacylglycerol (C36:4) | 639.4946 | 9.408274 | Robinson et al., 2018 |
| **34** | Diacylglycerol (C36:3) | 641.5112 | 9.9381895 | Robinson et al., 2018 |
| **35** | PC(30:0) | 706.541 | 8.492703 | (Alfano et al., 2019; Robinson et al., 2018), |
| **36** | PC(32:0) | 734.57 | 8.960004 | Robinson et al., 2018 |
| **37** | PC(34:2) | 758.5747 | 8.684198 | Robinson et al., 2018 |
| **38** | PlasmalogenPC(36:4) or PC(O-36:5) | 766.5815 | 8.858829 | Alfano et al., 2019; Robinson et al., 2018 |
| **39** | PlasmalogenPC(36:3) or PC(O-36:4) | 768.5883 | 9.189 | (Alfano et al., 2019; Robinson et al., 2018), |
| **40** | PC(36:4) | 782.5722 | 9.57233 | (Alfano et al., 2019; Robinson et al., 2018), |
| **41** | PC(36:4) isomer | 793.5614 | 8.628368 | Robinson et al., 2018 |
| **42** | Plasmalogen PC(38:4) or PC(O-38:5) | 794.6046 | 9.77853 | (Alfano et al., 2019; Robinson et al., 2018), |
| **43** | PC(38:4) | 810.6053 | 9.168946 | Robinson et al., 2018 |

Table S5: Logistic regression odds ratio per standard deviation (95% CI) for rapid growth at twelve months for Model 1* and 2** for the birthweight related metabolites.

| num | Metabolite | Model 1* | | | Model 2** | | |
| --- | --- | --- | --- | --- | --- | --- | --- |
|  |  | **Odd ratio (95%CIs)** | **p-value** | **False discovery rate** | **Odd ratio (95%CIs)** | **p-value** | **False discovery rate** |
| 1 | Butyrylcarnitine/Isobutyrylcarnitine (C4:0) | 1.231 (0.982,1.543) | 7.09E-02 | 1.42E-01 | 1.133 (0.855,1.502) | 3.84E-01 | 5.82E-01 |
| 2 | Decanoylcarnitine (C10:0) | 1.193 (0.945,1.506) | 1.37E-01 | 2.37E-01 | 1.204 (0.915,1.584) | 1.85E-01 | 3.95E-01 |
| 3 | Decenoylcarnitine (C10:1) | 1.405 (1.106,1.785) | 5.39E-03 | 2.32E-02 | 1.435 (1.087,1.893) | 1.07E-02 | 1.18E-01 |
| 4 | Dodecanoylcarnitine (C12:0) | 1.140 (0.907,1.432) | 2.61E-01 | 3.96E-01 | 1.195 (0.912,1.566) | 1.96E-01 | 3.95E-01 |
| 5 | Dodecenoylcarnitine (C12:1) | 1.121 (0.896,1.403) | 3.17E-01 | 4.65E-01 | 1.151 (0.882,1.502) | 3.02E-01 | 5.10E-01 |
| 6 | Hexadecadienoylcarnitine (C16:2) | 1.247 (0.998,1.557) | 5.21E-02 | 1.15E-01 | 1.138 (0.874,1.482) | 3.36E-01 | 5.27E-01 |
| 7 | Hexadecenoylcarnitine (C16:1) | 1.068 (0.852,1.338) | 5.68E-01 | 6.75E-01 | 1.060 (0.807,1.391) | 6.75E-01 | 7.56E-01 |
| 8 | Hexenoylcarnitine (C6:1) | 1.261 (0.984,1.617) | 6.73E-02 | 1.41E-01 | 1.088 (0.808,1.465) | 5.77E-01 | 7.27E-01 |
| 9 | Hydroxyhexadecadienoylcarnitine (C16:1-OH) | 1.180 (0.950,1.466) | 1.35E-01 | 2.37E-01 | 1.167 (0.901,1.512) | 2.41E-01 | 4.61E-01 |
| 10 | Hydroxytetradecenoylcarnitine (C14:1-OH) | 1.088 (0.868,1.364) | 4.63E-01 | 6.17E-01 | 1.071 (0.816,1.405) | 6.21E-01 | 7.39E-01 |
| 11 | Octanoylcarnitine (C8:0) | 1.155 (0.918,1.454) | 2.18E-01 | 3.42E-01 | 1.073 (0.822,1.400) | 6.05E-01 | 7.39E-01 |
| 12 | Tetradecadiencarnitine (C14:2) | 1.325 (1.059,1.658) | 1.39E-02 | 4.37E-02 | 1.315 (1.013,1.706) | 3.97E-02 | 2.01E-01 |
| 13 | Tetradecanoylcarnitine (C14:0) | 1.034 (0.816,1.311) | 7.80E-01 | 8.37E-01 | 0.991 (0.745,1.318) | 9.50E-01 | 9.50E-01 |
| 14 | Tetradecenoylcarnitine (C14:1) | 1.058 (0.845,1.324) | 6.26E-01 | 7.25E-01 | 1.048 (0.804,1.366) | 7.28E-01 | 7.81E-01 |
| 15 | Leucine | 1.045 (0.821,1.329) | 7.20E-01 | 7.92E-01 | 0.902 (0.678,1.200) | 4.79E-01 | 6.40E-01 |
| 16 | Tryptophan | 0.923 (0.736,1.158) | 4.89E-01 | 6.25E-01 | 0.947 (0.725,1.236) | 6.87E-01 | 7.56E-01 |
| 17 | Sphingosine | 0.942 (0.718,1.236) | 6.65E-01 | 7.50E-01 | 0.788 (0.605,1.028) | 7.94E-02 | 2.91E-01 |
| 18 | Docosahexaenoic acid | 1.292 (1.005,1.660) | 4.58E-02 | 1.06E-01 | 1.456 (1.109,1.911) | 6.78E-03 | 9.95E-02 |
| 19 | Diacylglycerol (C34:2) | 1.033 (0.778,1.372) | 8.20E-01 | 8.39E-01 | 1.135 (0.829,1.554) | 4.30E-01 | 6.10E-01 |
| 20 | Diacylglycerol (C36:3) | 1.084 (0.832,1.413) | 5.48E-01 | 6.70E-01 | 1.273 (0.961,1.688) | 9.30E-02 | 3.11E-01 |
| 21 | Diacylglycerol (C36:4) | 1.123 (0.880,1.432) | 3.51E-01 | 4.98E-01 | 1.376 (1.054,1.796) | 1.91E-02 | 1.40E-01 |
| 22 | LysoPC(16:1) | 1.004 (0.803,1.256) | 9.69E-01 | 9.69E-01 | 0.798 (0.608,1.046) | 1.02E-01 | 3.11E-01 |
| 23 | LysoPC(18:1) | 1.185 (0.946,1.484) | 1.40E-01 | 2.37E-01 | 0.939 (0.717,1.229) | 6.45E-01 | 7.47E-01 |
| 24 | LysoPC(18:3) | 1.090 (0.871,1.365) | 4.53E-01 | 6.17E-01 | 0.963 (0.732,1.266) | 7.86E-01 | 8.24E-01 |
| 25 | LysoPC(20:2) | 1.303 (1.012,1.677) | 3.97E-02 | 9.71E-02 | 1.081 (0.822,1.420) | 5.78E-01 | 7.27E-01 |
| 26 | LysoPC(20:4) | 1.435 (1.106,1.861) | 6.52E-03 | 2.39E-02 | 1.282 (0.949,1.733) | 1.06E-01 | 3.11E-01 |
| 27 | LysoPC(22:5) | 0.971 (0.759,1.243) | 8.16E-01 | 8.39E-01 | 0.757 (0.564,1.016) | 6.33E-02 | 2.53E-01 |
| 28 | LysoPC(22:6) | 0.862 (0.682,1.090) | 2.14E-01 | 3.42E-01 | 0.857 (0.657,1.118) | 2.55E-01 | 4.67E-01 |
| 29 | PC(30:0) | 1.312 (1.046,1.647) | 1.89E-02 | 5.55E-02 | 0.987 (0.749,1.302) | 9.29E-01 | 9.50E-01 |
| 30 | PC(32:0) | 1.396 (1.112,1.753) | 4.06E-03 | 2.20E-02 | 1.148 (0.885,1.488) | 2.99E-01 | 5.10E-01 |
| 31 | PC(34:2) | 1.542 (1.222,1.946) | 2.62E-04 | 3.84E-03 | 1.456 (1.132,1.872) | 3.46E-03 | 7.62E-02 |
| 32 | PC(36:4) | 1.415 (1.125,1.779) | 2.99E-03 | 2.19E-02 | 1.119 (0.856,1.462) | 4.11E-01 | 6.03E-01 |
| 33 | PC(36:4) isomer | 1.417 (1.114,1.802) | 4.49E-03 | 2.20E-02 | 1.297 (0.988,1.702) | 6.06E-02 | 2.53E-01 |
| 34 | PC(38:4) | 1.274 (1.019,1.593) | 3.33E-02 | 9.15E-02 | 1.197 (0.928,1.544) | 1.67E-01 | 3.86E-01 |
| 35 | Plasmalogen PC(38:4) or PC(O-38:5) | 1.379 (1.097,1.732) | 5.80E-03 | 2.32E-02 | 1.099 (0.846,1.429) | 4.80E-01 | 6.40E-01 |
| 36 | PlasmalogenPC(36:3) or PC(O-36:4) | 1.431 (1.142,1.792) | 1.84E-03 | 1.62E-02 | 1.218 (0.943,1.572) | 1.31E-01 | 3.19E-01 |
| 37 | PlasmalogenPC(36:4) or PC(O-36:5) | 1.484 (1.180,1.868) | 7.52E-04 | 8.27E-03 | 1.317 (1.011,1.716) | 4.11E-02 | 2.01E-01 |
| 38 | Cholestenone | 2.064 (1.594,2.673) | 3.98E-08 | 1.75E-06 | 1.755 (1.236,2.491) | 1.66E-03 | 3.32E-03 |
| 39 | Cholesterol | 1.535 (1.220,1.930) | 2.49E-04 | 3.84E-03 | 1.229 (0.941,1.606) | 1.30E-01 | 3.19E-01 |
| 40 | Progesterone | 1.402 (1.092,1.800) | 7.97E-03 | 2.70E-02 | 1.428 (1.051,1.940) | 2.26E-02 | 1.42E-01 |
| 41 | Indolelactic acid | 1.300 (1.013,1.667) | 3.89E-02 | 9.71E-02 | 1.255 (0.938,1.679) | 1.26E-01 | 3.19E-01 |
| 42 | Methoxykynurenic acid | 1.084 (0.859,1.367) | 4.98E-01 | 6.25E-01 | 1.150 (0.867,1.524) | 3.33E-01 | 5.27E-01 |
| 43 | Retinol | 0.792 (0.603,1.041) | 9.41E-02 | 1.80E-01 | 0.820 (0.606,1.109) | 1.97E-01 | 3.95E-01 |

**** Model 1 (adjusted for sex and age of child at outcome measurement, ethnicity and we used a random effects model by cohort)***

*****Model 2 (Model 1 adjusted for maternal BMI, paternal BMI, gestational age, weight gained during pregnancy, paternal education, passive and active smoking status during pregnancy, parity, and mode of delivery)***

Table S6: All metabolomic features significantly associated (FDR 5%) with overweight/obesity at early childhood. In case of more than one feature per compound were detected, the feature with highest intensity is written in bold.

| **Compound** | ***m/z*** | **Rt(min)** | **Annotation** | **Estimate** | **Std Error** | **t-value** | **p-value*** |
| --- | --- | --- | --- | --- | --- | --- | --- |
| **5** | **129.0025** | **0.4939376** | **Unidentified (U1)** | **-0.661** | **0.185** | **-3.571** | **3.55E-04** |
| 5 | 86.99288 | 0.4942084 | Unidentified (U1) | -0.627 | 0.179 | -3.512 | 4.44E-04 |
|  |  |  |  |  |  |  |  |
| **6** | **196.9619** | **0.5246815** | **Unidentified (U2)** | **0.832** | **0.219** | **3.794** | **1.48E-04** |
| 6 | 253.9104 | 0.5244932 | Unidentified (U2) | 0.762 | 0.214 | 3.565 | 3.64E-04 |
|  |  |  |  |  |  |  |  |
| 7 | 514.878 | 0.5776492 | Unidentified (U3) | -0.698 | 0.170 | -4.115 | 3.88E-05 |
| 7 | 582.8643 | 0.5770611 | Unidentified (U3) | -0.677 | 0.165 | -4.111 | 3.94E-05 |
| 7 | 446.8882 | 0.577687 | Unidentified (U3) | -0.676 | 0.165 | -4.100 | 4.14E-05 |
| 7 | 700.8185 | 0.5779851 | Unidentified (U3) | -0.767 | 0.189 | -4.065 | 4.81E-05 |
| 7 | 378.9011 | 0.5771529 | Unidentified (U3) | -0.683 | 0.168 | -4.063 | 4.85E-05 |
| 7 | 726.8223 | 0.5750447 | Unidentified (U3) | -0.720 | 0.179 | -4.032 | 5.53E-05 |
| 7 | 760.8202 | 0.5745219 | Unidentified (U3) | -0.684 | 0.170 | -4.020 | 5.82E-05 |
| 7 | 650.8544 | 0.5757769 | Unidentified (U3) | -0.612 | 0.154 | -3.981 | 6.85E-05 |
| 7 | 692.8324 | 0.5753962 | Unidentified (U3) | -0.723 | 0.182 | -3.973 | 7.11E-05 |
| 7 | 718.8356 | 0.5748507 | Unidentified (U3) | -0.574 | 0.147 | -3.914 | 9.09E-05 |
| **7** | **242.9253** | **0.5751633** | **Unidentified (U3)** | **-0.696** | **0.178** | **-3.906** | **9.39E-05** |
| 7 | 312.9127 | 0.5741116 | Unidentified (U3) | -0.714 | 0.183 | -3.903 | 9.49E-05 |
| 7 | 108.9488 | 0.5738347 | Unidentified (U3) | -0.724 | 0.187 | -3.866 | 1.11E-04 |
| 7 | 106.9512 | 0.5741819 | Unidentified (U3) | -0.713 | 0.187 | -3.821 | 1.33E-04 |
| 7 | 310.9139 | 0.5743221 | Unidentified (U3) | -0.689 | 0.180 | -3.819 | 1.34E-04 |
| 7 | 658.8351 | 0.5753322 | Unidentified (U3) | -0.704 | 0.185 | -3.809 | 1.40E-04 |
| 7 | 176.937 | 0.5720361 | Unidentified (U3) | -0.716 | 0.189 | -3.796 | 1.47E-04 |
| 7 | 174.9394 | 0.5723583 | Unidentified (U3) | -0.707 | 0.187 | -3.771 | 1.62E-04 |
| 7 | 828.8043 | 0.5720578 | Unidentified (U3) | -0.645 | 0.175 | -3.696 | 2.19E-04 |
| 7 | 870.7891 | 0.5769692 | Unidentified (U3) | -0.710 | 0.196 | -3.629 | 2.84E-04 |
| 7 | 624.843 | 0.5741374 | Unidentified (U3) | -0.726 | 0.200 | -3.620 | 2.94E-04 |
| 7 | 598.8356 | 0.5796612 | Unidentified (U3) | -0.705 | 0.195 | -3.620 | 2.95E-04 |
| 7 | 462.8653 | 0.5798938 | Unidentified (U3) | -0.695 | 0.194 | -3.581 | 3.42E-04 |
| 7 | 394.8761 | 0.5797012 | Unidentified (U3) | -0.695 | 0.195 | -3.571 | 3.56E-04 |
| 7 | 530.8525 | 0.579978 | Unidentified (U3) | -0.694 | 0.195 | -3.565 | 3.63E-04 |
| 7 | 666.8233 | 0.5794433 | Unidentified (U3) | -0.712 | 0.200 | -3.557 | 3.75E-04 |
| 7 | 258.9015 | 0.5785645 | Unidentified (U3) | -0.682 | 0.193 | -3.530 | 4.15E-04 |
| 7 | 794.8107 | 0.5737721 | Unidentified (U3) | -0.611 | 0.173 | -3.529 | 4.16E-04 |
| 7 | 734.812 | 0.5787313 | Unidentified (U3) | -0.696 | 0.197 | -3.527 | 4.21E-04 |
| 7 | 326.8868 | 0.5771512 | Unidentified (U3) | -0.686 | 0.196 | -3.494 | 4.76E-04 |
|  |  |  |  |  |  |  |  |
| 8 | 154.0264 | 0.6849625 | Unidentified (U4) | -0.702 | 0.172 | -4.088 | 4.35E-05 |
|  |  |  |  |  |  |  |  |
| 9 | 169.134 | 0.6985534 | Unidentified (U5) | -0.759 | 0.206 | -3.687 | 2.27E-04 |
|  |  |  |  |  |  |  |  |
| 10 | 209.1159 | 6.164805 | Unidentified (U7) | -0.671 | 0.181 | -3.705 | 2.12E-04 |
|  |  |  |  |  |  |  |  |
| 13 | 443.4095 | 8.544215 | Unidentified (U9) | 0.893 | 0.221 | 4.046 | 5.21E-05 |
| **13** | **460.4366** | **8.543666** | **Unidentified (U9)** | **1.001** | **0.262** | **3.822** | **1.32E-04** |
|  |  |  |  |  |  |  |  |
| **14** | **72.08108** | **0.8007007** | **Valine** | **-0.611** | **0.163** | **-3.748** | **1.78E-04** |
| 14 | 249.0292 | 0.8028366 | Valine | -0.670 | 0.181 | -3.694 | 2.21E-04 |

*Model was adjusted for child’s sex and age at outcome measurement and ethnicity. We used a random effects model by cohort

Table S7: Logistic regression odds ratio per standard deviation (95% CI) for overweight/obesity in early childhood for Model 1* and 2** for the birthweight related metabolites.

| num | Metabolite | Model 1*** | | | Model 2**** | | |
| --- | --- | --- | --- | --- | --- | --- | --- |
|  |  | **Odd ratio (95%CIs)** | **p-value** | **False discovery rate** | **Odd ratio (95%CIs)** | **p-value** | **False discovery rate** |
| 1 | Butyrylcarnitine/Isobutyrylcarnitine (C4:0) | 0.777 (0.570,1.058) | 1.09E-01 | 6.43E-01 | 0.734 (0.498,1.081) | 1.17E-01 | 3.58E-01 |
| 2 | Decanoylcarnitine (C10:0) | 0.912 (0.653,1.274) | 5.89E-01 | 8.36E-01 | 0.868 (0.581,1.296) | 4.88E-01 | 7.67E-01 |
| 3 | Decenoylcarnitine (C10:1) | 1.193 (0.858,1.659) | 2.93E-01 | 7.70E-01 | 1.155 (0.769,1.734) | 4.88E-01 | 7.67E-01 |
| 4 | Dodecanoylcarnitine (C12:0) | 0.906 (0.646,1.272) | 5.70E-01 | 8.36E-01 | 0.834 (0.553,1.258) | 3.86E-01 | 7.04E-01 |
| 5 | Dodecenoylcarnitine (C12:1) | 0.902 (0.658,1.235) | 5.20E-01 | 8.36E-01 | 0.716 (0.476,1.077) | 1.09E-01 | 3.58E-01 |
| 6 | Hexadecadienoylcarnitine (C16:2) | 0.941 (0.701,1.263) | 6.86E-01 | 8.39E-01 | 0.928 (0.649,1.326) | 6.80E-01 | 9.07E-01 |
| 7 | Hexadecenoylcarnitine (C16:1) | 0.772 (0.544,1.094) | 1.46E-01 | 6.43E-01 | 0.627 (0.400,0.984) | 4.23E-02 | 3.12E-01 |
| 8 | Hexenoylcarnitine (C6:1) | 0.764 (0.530,1.102) | 1.50E-01 | 6.43E-01 | 0.613 (0.382,0.984) | 4.26E-02 | 3.12E-01 |
| 9 | Hydroxyhexadecadienoylcarnitine (C16:1-OH) | 1.033 (0.787,1.356) | 8.14E-01 | 9.01E-01 | 1.040 (0.746,1.449) | 8.18E-01 | 9.66E-01 |
| 10 | Hydroxytetradecenoylcarnitine (C14:1-OH) | 1.096 (0.801,1.498) | 5.68E-01 | 8.36E-01 | 1.053 (0.711,1.559) | 7.96E-01 | 9.66E-01 |
| 11 | Octanoylcarnitine (C8:0) | 0.963 (0.695,1.333) | 8.19E-01 | 9.01E-01 | 0.936 (0.643,1.363) | 7.31E-01 | 9.28E-01 |
| 12 | Tetradecadiencarnitine (C14:2) | 1.004 (0.744,1.355) | 9.79E-01 | 9.79E-01 | 0.940 (0.653,1.352) | 7.39E-01 | 9.28E-01 |
| 13 | Tetradecanoylcarnitine (C14:0) | 0.880 (0.638,1.214) | 4.37E-01 | 8.36E-01 | 0.776 (0.531,1.133) | 1.88E-01 | 4.22E-01 |
| 14 | Tetradecenoylcarnitine (C14:1) | 0.907 (0.657,1.251) | 5.51E-01 | 8.36E-01 | 0.779 (0.523,1.160) | 2.19E-01 | 4.37E-01 |
| 15 | Leucine | 0.658 (0.460,0.941) | 2.17E-02 | 4.78E-01 | 0.469 (0.293,0.751) | 1.61E-03 | 4.90E-02 |
| 16 | Tryptophan | 0.728 (0.529,1.002) | 5.11E-02 | 6.28E-01 | 0.720 (0.496,1.045) | 8.38E-02 | 3.58E-01 |
| 17 | Sphingosine | 0.821 (0.552,1.221) | 3.29E-01 | 7.70E-01 | 0.742 (0.507,1.086) | 1.25E-01 | 3.58E-01 |
| 18 | Docosahexaenoic acid | 0.610 (0.412,0.903) | 1.35E-02 | 4.78E-01 | 0.619 (0.395,0.970) | 3.63E-02 | 3.12E-01 |
| 19 | Diacylglycerol (C34:2) | 0.835 (0.537,1.300) | 4.25E-01 | 8.36E-01 | 0.640 (0.372,1.100) | 1.07E-01 | 3.58E-01 |
| 20 | Diacylglycerol (C36:3) | 0.920 (0.618,1.368) | 6.79E-01 | 8.39E-01 | 0.990 (0.654,1.498) | 9.63E-01 | 9.93E-01 |
| 21 | Diacylglycerol (C36:4) | 1.146 (0.820,1.601) | 4.24E-01 | 8.36E-01 | 1.182 (0.801,1.742) | 4.00E-01 | 7.04E-01 |
| 22 | LysoPC(16:1) | 1.213 (0.880,1.671) | 2.38E-01 | 7.50E-01 | 1.154 (0.789,1.688) | 4.59E-01 | 7.67E-01 |
| 23 | LysoPC(18:1) | 1.117 (0.813,1.536) | 4.94E-01 | 8.36E-01 | 1.118 (0.764,1.636) | 5.65E-01 | 7.94E-01 |
| 24 | LysoPC(18:3) | 1.253 (0.917,1.713) | 1.56E-01 | 6.43E-01 | 1.263 (0.875,1.821) | 2.12E-01 | 4.37E-01 |
| 25 | LysoPC(20:2) | 1.022 (0.704,1.484) | 9.09E-01 | 9.46E-01 | 1.126 (0.784,1.618) | 5.20E-01 | 7.88E-01 |
| 26 | LysoPC(20:4) | 0.959 (0.689,1.334) | 8.03E-01 | 9.01E-01 | 0.752 (0.520,1.088) | 1.30E-01 | 3.58E-01 |
| 27 | LysoPC(22:5) | 1.102 (0.759,1.601) | 6.10E-01 | 8.38E-01 | 0.957 (0.593,1.545) | 8.58E-01 | 9.68E-01 |
| 28 | LysoPC(22:6) | 0.928 (0.664,1.297) | 6.61E-01 | 8.39E-01 | 0.993 (0.706,1.399) | 9.70E-01 | 9.93E-01 |
| 29 | PC(30:0) | 0.968 (0.702,1.335) | 8.42E-01 | 9.04E-01 | 1.043 (0.702,1.551) | 8.34E-01 | 9.66E-01 |
| 30 | PC(32:0) | 1.015 (0.746,1.381) | 9.25E-01 | 9.46E-01 | 1.115 (0.760,1.637) | 5.78E-01 | 7.94E-01 |
| 31 | PC(34:2) | 1.068 (0.775,1.472) | 6.87E-01 | 8.39E-01 | 1.271 (0.887,1.821) | 1.92E-01 | 4.22E-01 |
| 32 | PC(36:4) | 0.905 (0.661,1.238) | 5.31E-01 | 8.36E-01 | 0.981 (0.680,1.414) | 9.17E-01 | 9.84E-01 |
| 33 | PC(36:4) isomer | 0.829 (0.616,1.115) | 2.14E-01 | 7.24E-01 | 0.750 (0.527,1.067) | 1.10E-01 | 3.58E-01 |
| 34 | PC(38:4) | 0.810 (0.594,1.104) | 1.81E-01 | 6.65E-01 | 0.717 (0.486,1.057) | 9.34E-02 | 3.58E-01 |
| 35 | Plasmalogen PC(38:4) or PC(O-38:5) | 0.795 (0.576,1.096) | 1.61E-01 | 6.43E-01 | 0.762 (0.522,1.115) | 1.61E-01 | 4.18E-01 |
| 36 | PlasmalogenPC(36:3) or PC(O-36:4) | 0.855 (0.623,1.173) | 3.33E-01 | 7.70E-01 | 0.809 (0.556,1.178) | 2.69E-01 | 5.15E-01 |
| 37 | PlasmalogenPC(36:4) or PC(O-36:5) | 0.796 (0.582,1.088) | 1.53E-01 | 6.43E-01 | 0.764 (0.519,1.125) | 1.73E-01 | 4.22E-01 |
| 38 | Cholestenone | 1.312 (0.942,1.826) | 1.08E-01 | 6.43E-01 | 1.343 (0.923,1.953) | 1.23E-01 | 3.58E-01 |
| 39 | Cholesterol | 0.919 (0.675,1.250) | 5.89E-01 | 8.36E-01 | 0.999 (0.691,1.445) | 9.97E-01 | 9.97E-01 |
| 40 | Progesterone | 0.732 (0.531,1.009) | 5.71E-02 | 6.28E-01 | 0.590 (0.383,0.910) | 1.71E-02 | 2.81E-01 |
| 41 | Indolelactic acid | 0.854 (0.629,1.160) | 3.13E-01 | 7.70E-01 | 0.657 (0.462,0.934) | 1.92E-02 | 2.81E-01 |
| 42 | Methoxykynurenic acid | 1.185 (0.843,1.667) | 3.29E-01 | 7.70E-01 | 0.977 (0.656,1.456) | 9.10E-01 | 9.84E-01 |
| 43 | Retinol | 0.866 (0.590,1.271) | 4.62E-01 | 8.36E-01 | 0.731 (0.488,1.094) | 1.27E-01 | 3.58E-01 |

**** Model 1 (adjusted for child sex and age at outcome measurement, ethnicity and we used a random effects model by cohort),***

*****Model 2 (Model 1 adjusted for maternal BMI, paternal BMI, gestational age, weight gained during pregnancy, paternal education, passive and active smoking status during pregnancy, parity, and mode of delivery)***


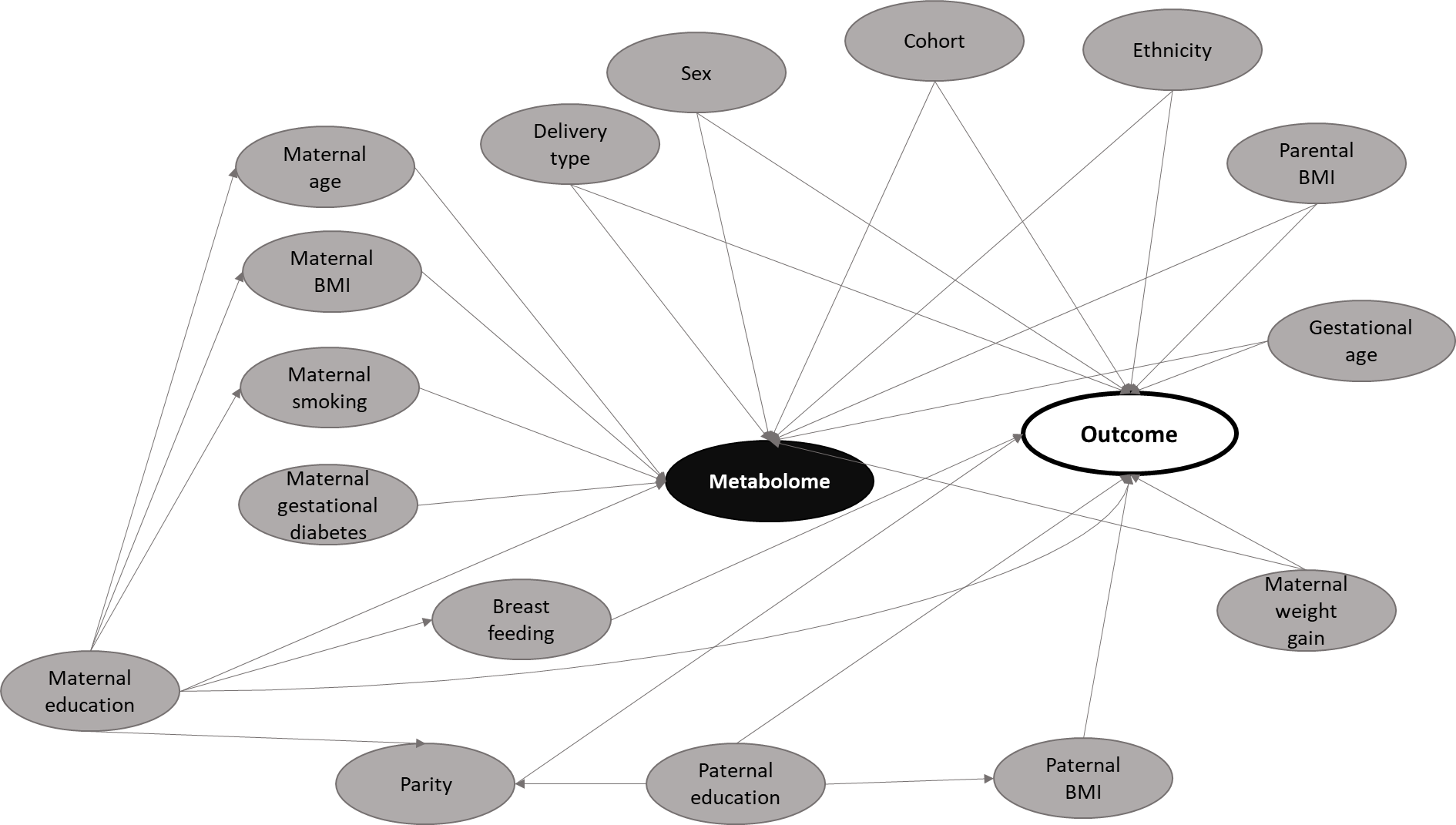


Figure S1: Directed acyclical graph (DAG) to visualise assumptions regarding covariates, metabolome and outcome. Covariates are coloured gray, metabolome black and outcome white. The examining outcomes are: 1) rapid growth at 1^st^ year of age and 2) overweight/obesity in early childhood.


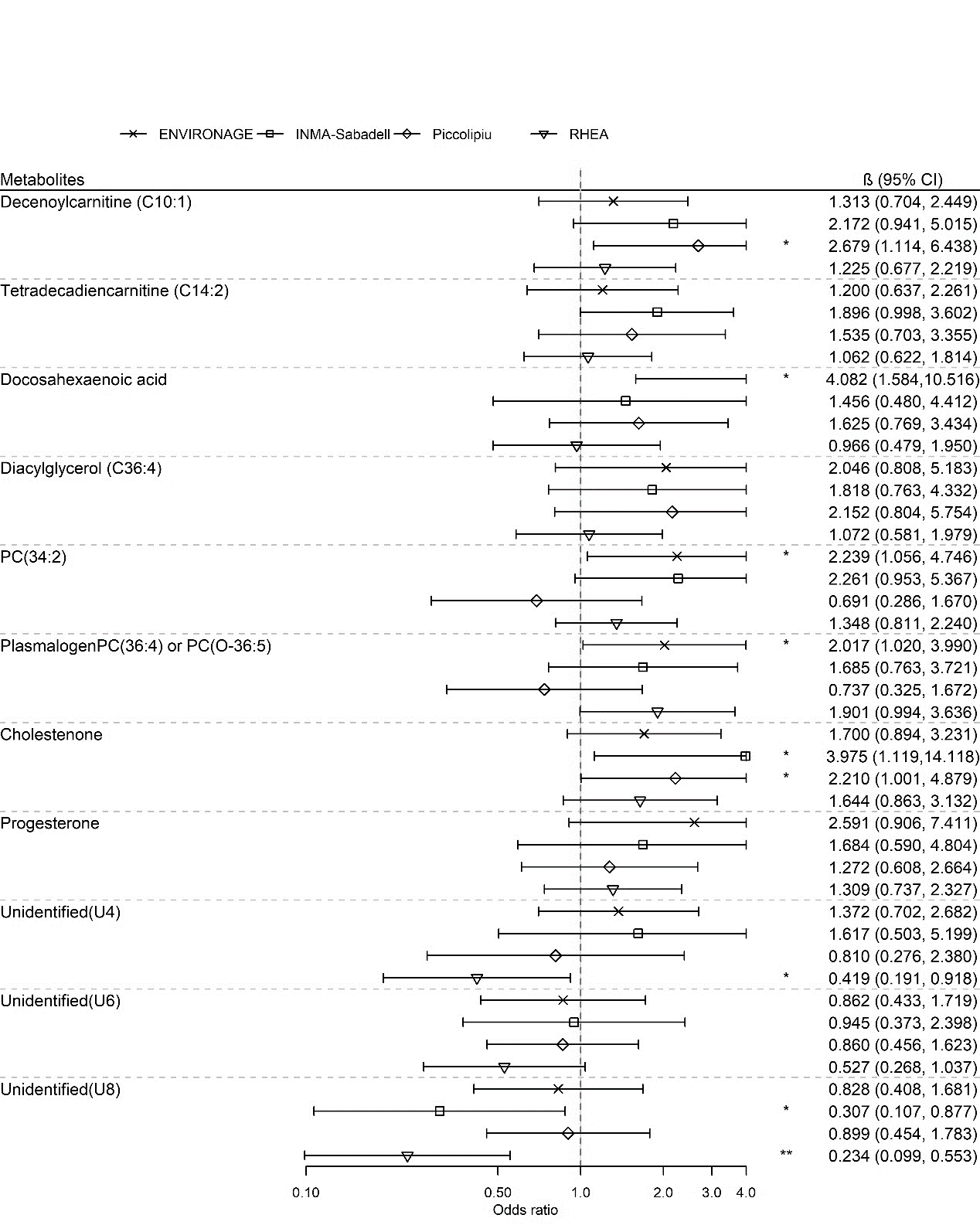


Figure S2: Logistic regression odds ratio per standard deviation (95% CI) for rapid growth for Model 2 (adjusted for child sex and age at outcome measurement, ethnicity and we used a random effects model by cohort adjusted for maternal BMI, paternal BMI, gestational age, weight gained during pregnancy, paternal education, passive and active smoking status during pregnancy, parity, and mode of delivery) stratified by cohort for the 8 between the 6 nominal statistically significant birthweight related metabolites and the 4 associated with rapid growth at 12 months. Where * is P< 0.05 and ** is FDR<0.05. Bars show 95% confidence intervals.


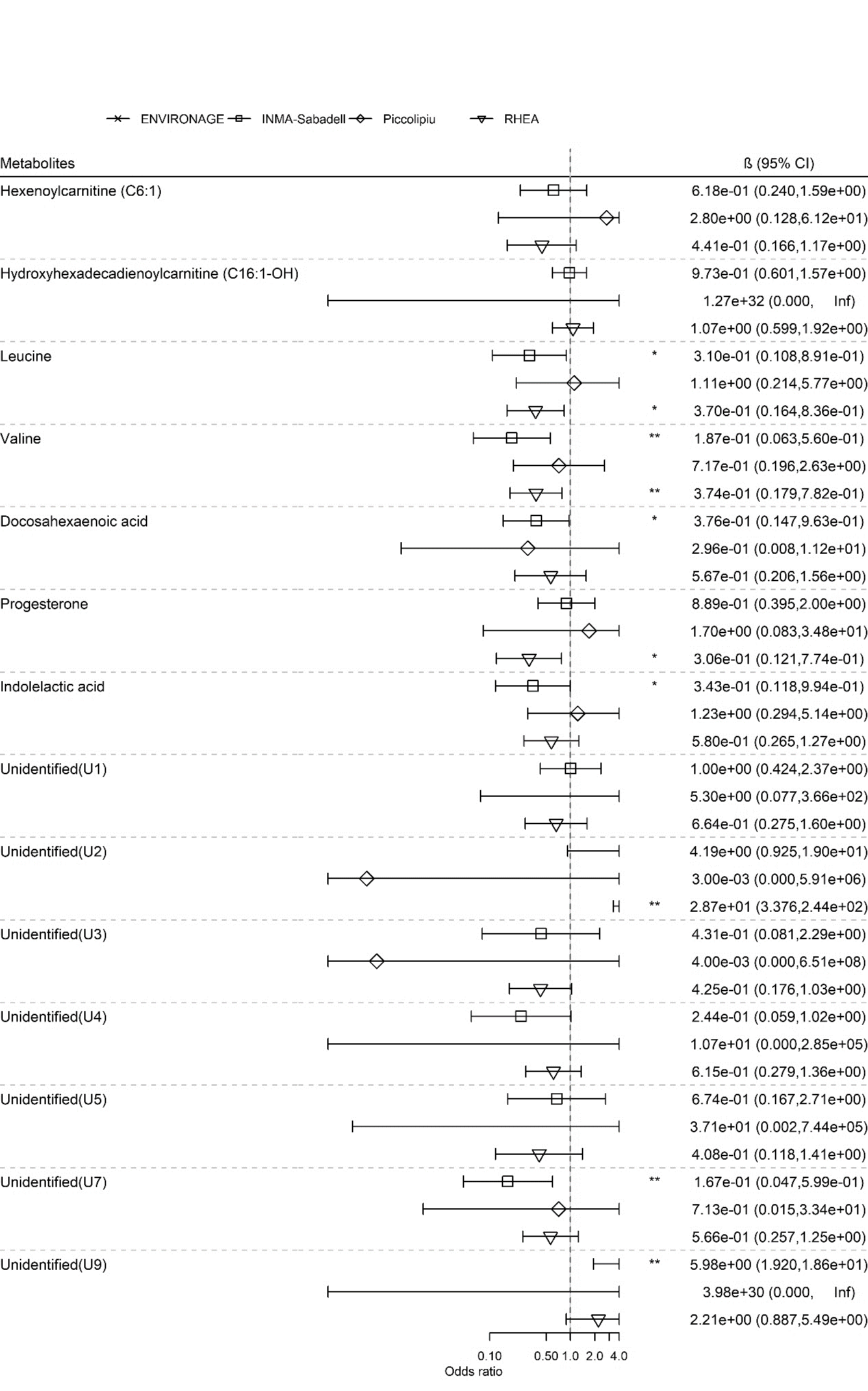


Figure S3: Logistic regression odds ratio per standard deviation (95% CI) for overweight/obesity in early childhood for Model 2 (adjusted for child sex and age at outcome measurement, ethnicity and we used a random effects model by cohort adjusted for maternal BMI, paternal BMI, gestational age, weight gained during pregnancy, paternal education, passive and active smoking status during pregnancy, parity, and mode of delivery) stratified by cohort for the 6 nominal statistically significant birthweight related metabolites and the 8 associated with overweight/obesity in early childhood. Where * is P< 0.05 and ** is FDR<0.05. Bars show 95% confidence intervals.


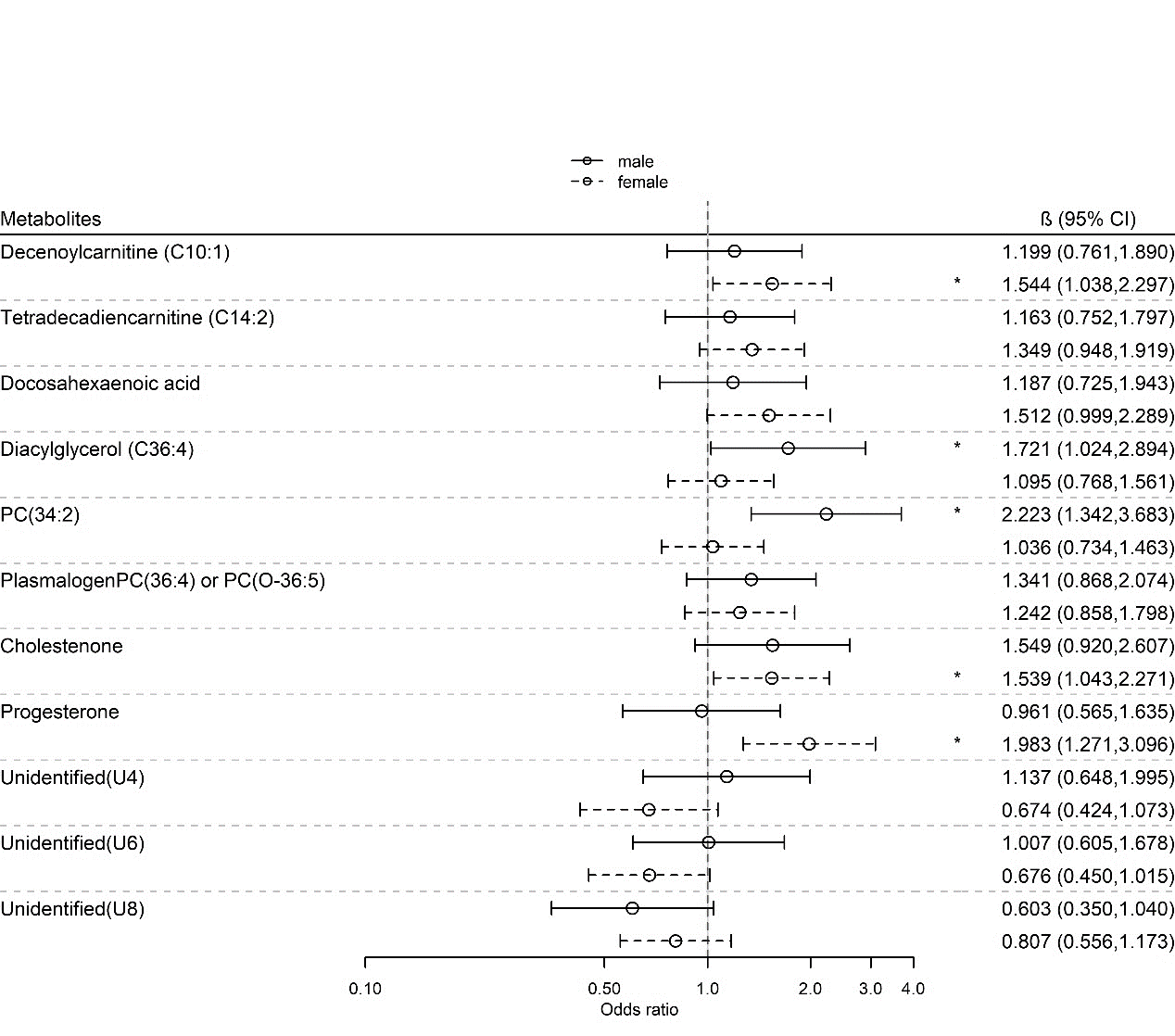


Figure S4: Logistic regression odds ratio per standard deviation (95% CI) for rapid growth for Model 2 (adjusted for child sex, age at outcome measurement, ethnicity and we used a random effects model by cohort adjusted for maternal BMI, paternal BMI, gestational age, weight gained during pregnancy, paternal education, passive and active smoking status during pregnancy, parity, and mode of delivery) stratified by sex for the 7 nominal statistically significant birthweight related metabolites and the 4 associated with rapid growth at 12 months. Where * is P< 0.05 and ** is FDR<0.05. Bars show 95% confidence intervals.


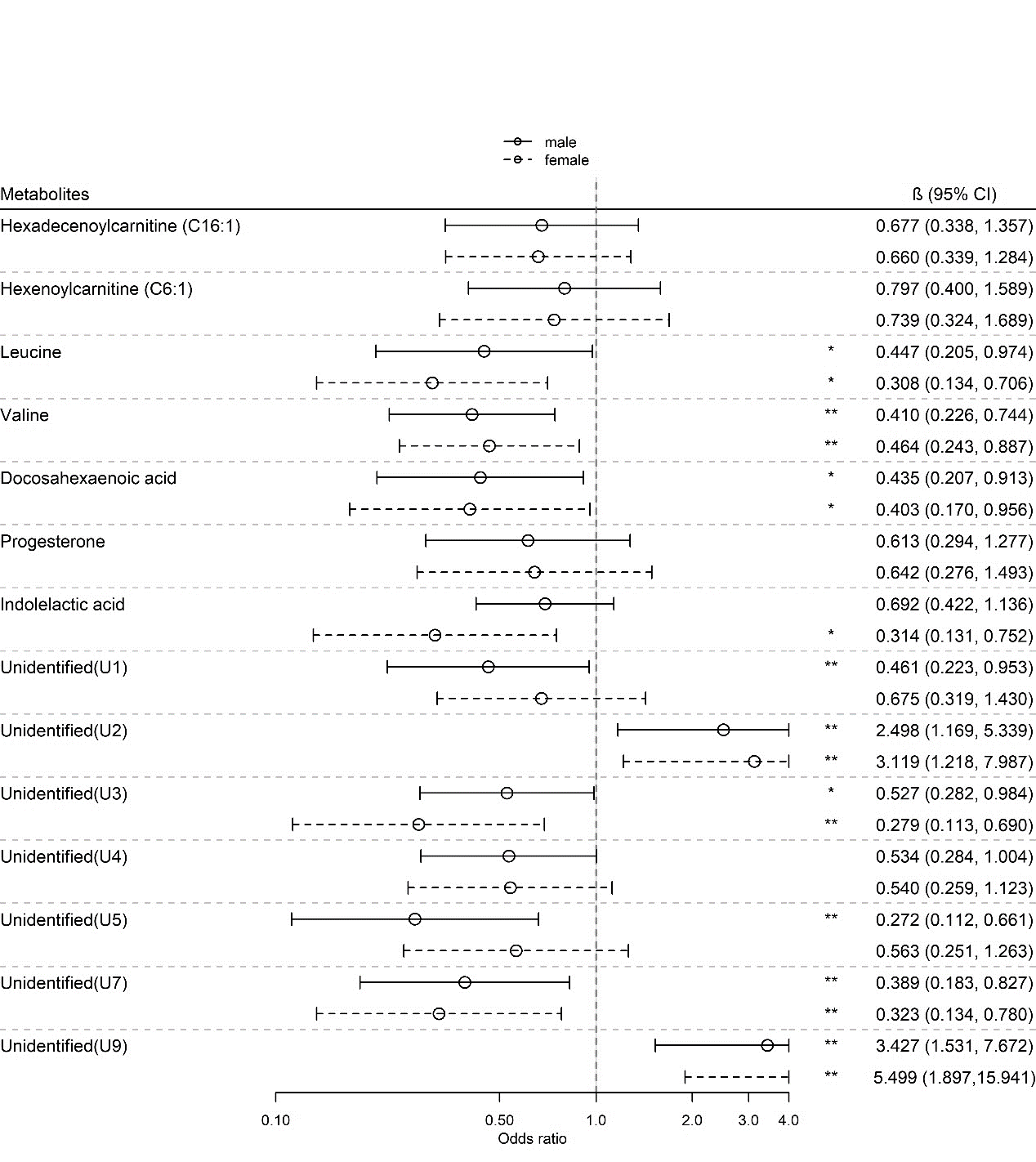


Figure S5: Logistic regression odds ratio per standard deviation (95% CI) for overweight/obesity in early childhood for Model 2 (adjusted for child sex, age at outcome measurement, ethnicity and we used a random effects model by cohort adjusted for maternal BMI, paternal BMI, gestational age, weight gained during pregnancy, paternal education, passive and active smoking status during pregnancy, parity, and mode of delivery) stratified by sex for the 6 nominal statistically significant birthweight related metabolites and the 8 associated with overweight/obesity in early childhood. Where * is P< 0.05 and ** is FDR<0.05. Bars show 95% confidence intervals.


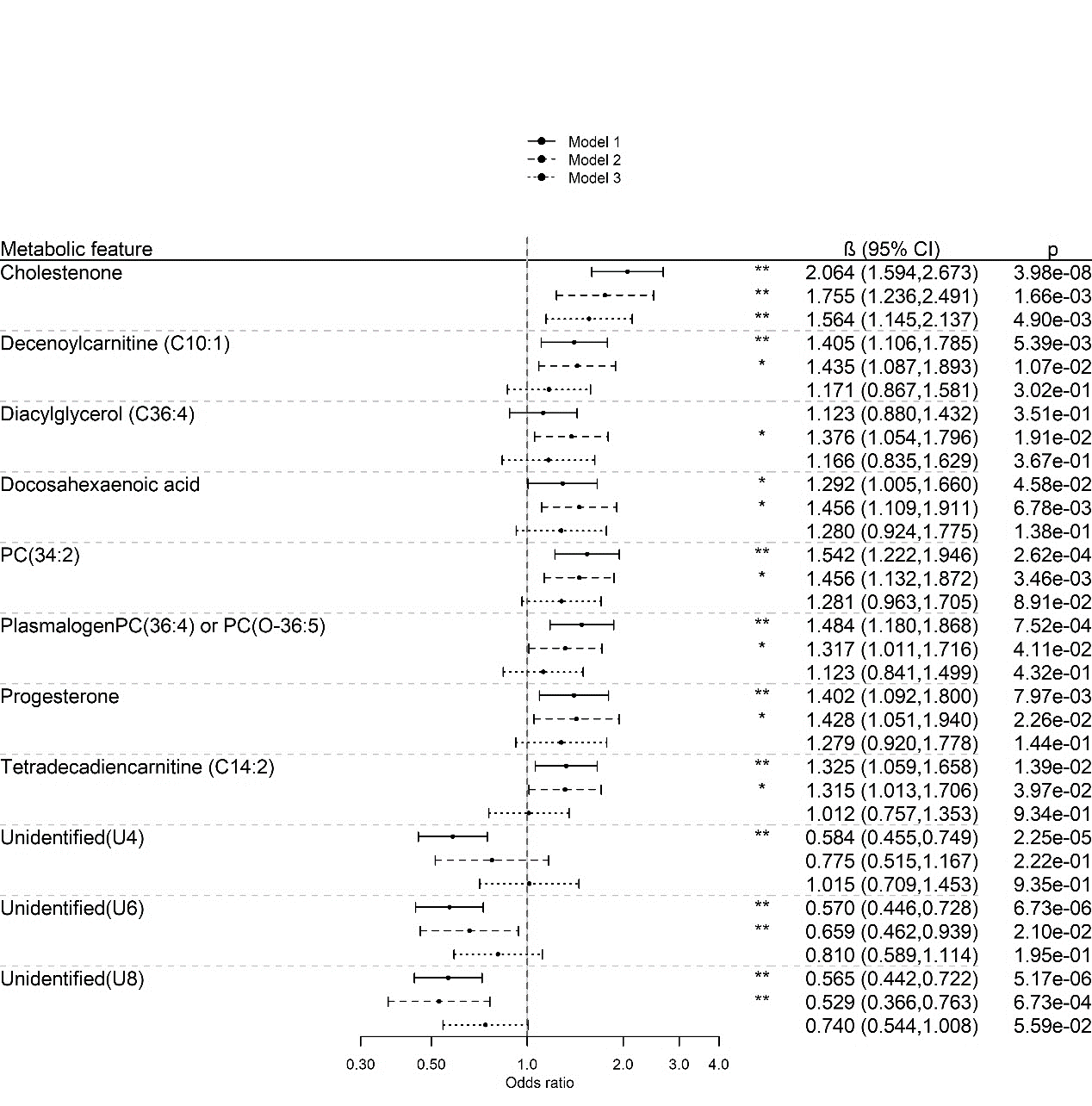


Figure S6: Logistic regression odds ratio per standard deviation (95% CI) for rapid growth at twelve months for Model 1 (adjusted for child sex and age at outcome measurement, ethnicity and we used a random effects model by cohort), Model 2 (Model 1 adjusted for maternal BMI, paternal BMI, gestational age, weight gained during pregnancy, paternal education, passive and active smoking status during pregnancy, parity, and mode of delivery) and Model 3 (Model 2 adjusted for birthweight) for the 7 nominal statistically significant birthweight related metabolites and the 4 associated with rapid growth at twelve months. Where * is P< 0.05 and ** is FDR<0.05. Bars show 95% confidence intervals.


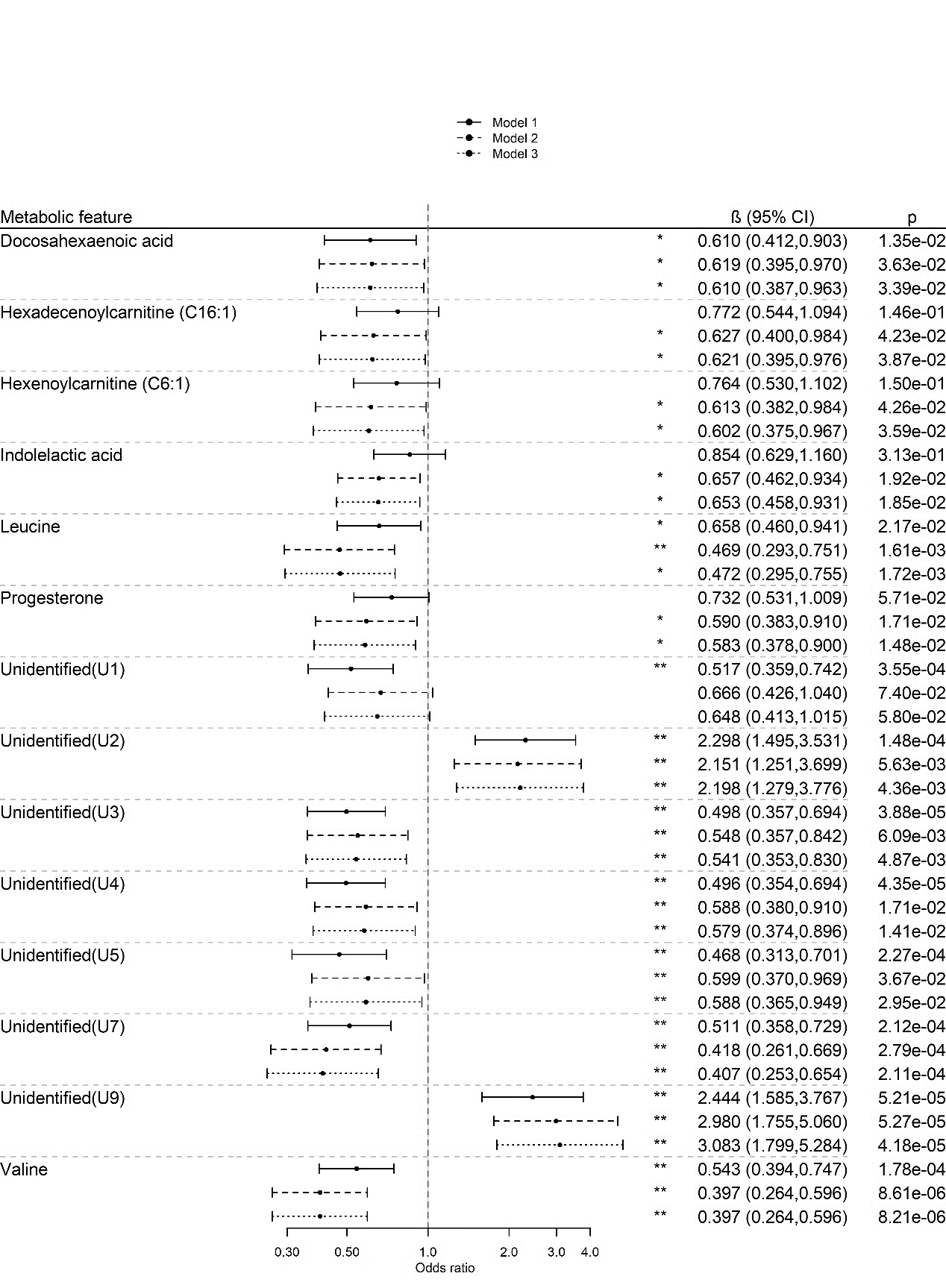


Figure S7: Logistic regression odds ratio per standard deviation (95% CI) for overweight/obesity in early childhood for Model 1 (adjusted for child sex and age at outcome measurement, ethnicity and we used a random effects model by cohort), Model 2 (Model 1 adjusted for maternal BMI, paternal BMI, gestational age, weight gained during pregnancy, paternal education, passive and active smoking status during pregnancy, parity, and mode of delivery) and Model 3 (Model 2 adjusted for birthweight) for the 6 nominal statistically significant birthweight related metabolites and the 8 associated with overweight/obesity in early childhood. Where * is P< 0.05 and ** is FDR<0.05. Bars show 95% confidence intervals.


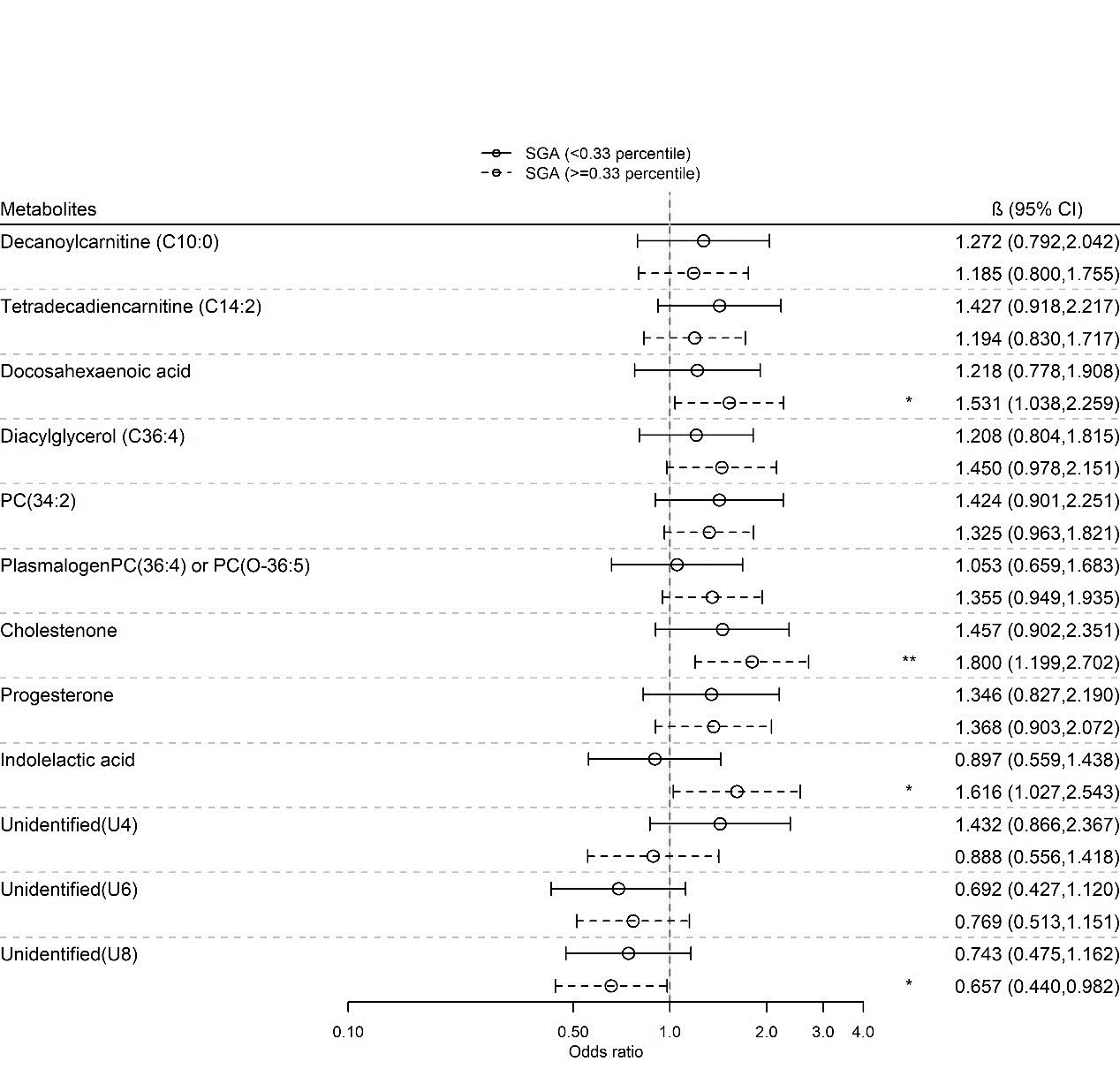


Figure S8: Logistic regression odds ratio per standard deviation (95% CI) for rapid growth for Model 2 (adjusted for child sex, age at outcome measurement, ethnicity and we used a random effects model by cohort adjusted for maternal BMI, paternal BMI, gestational age, weight gained during pregnancy, paternal education, passive and active smoking status during pregnancy, parity, and mode of delivery) stratified by Small of Gestational Age (SGA) for the 7 nominal statistically significant birthweight related metabolites and the 4 associated with rapid growth at 12 months. Where * is P< 0.05 and ** is FDR<0.05. Bars show 95% confidence intervals.


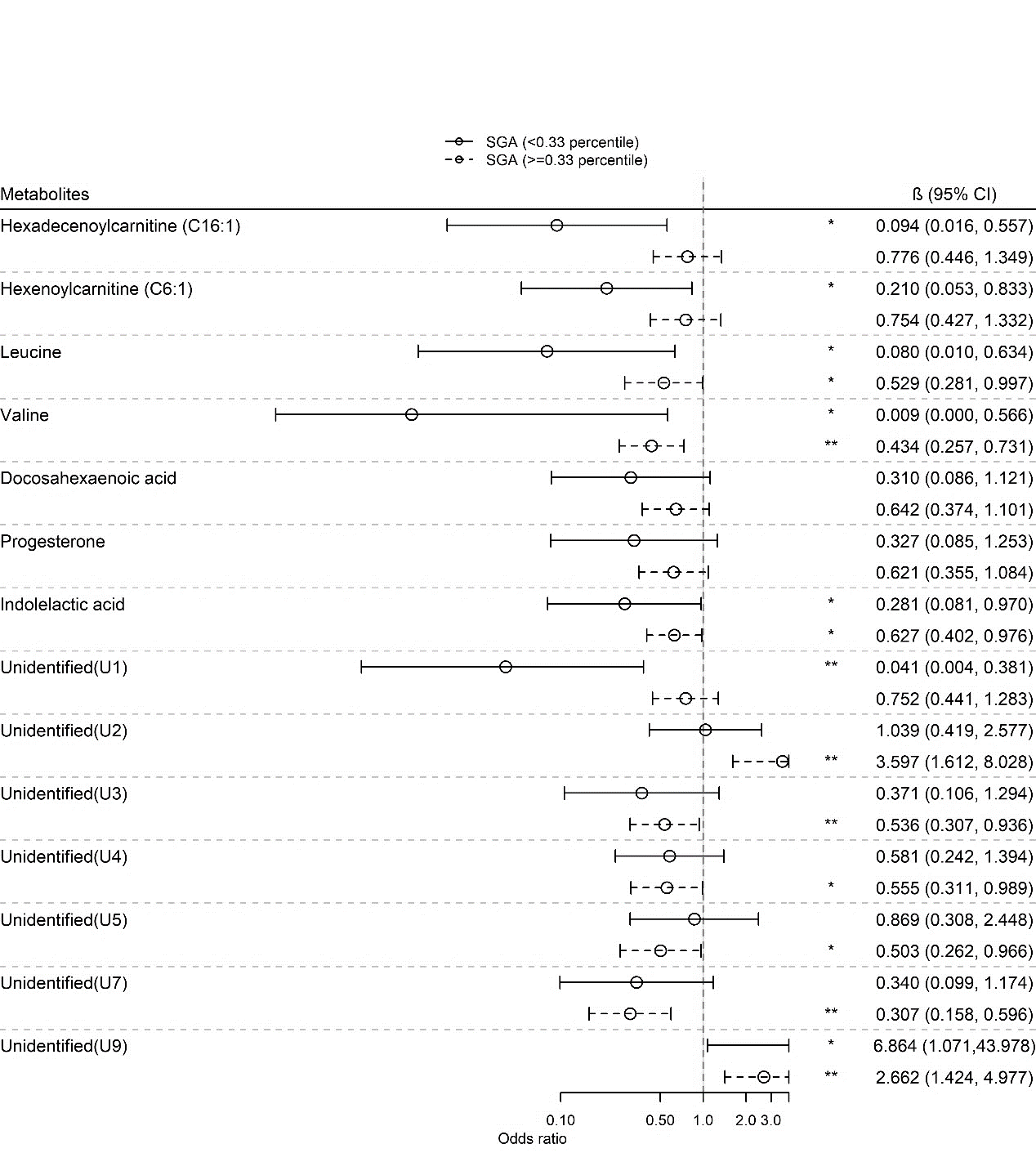


Figure S9: Logistic regression odds ratio per standard deviation (95% CI) for overweight/obesity in early childhood for Model 2 (adjusted for child sex, age at outcome measurement, ethnicity and we used a random effects model by cohort adjusted for maternal BMI, paternal BMI, gestational age, weight gained during pregnancy, paternal education, passive and active smoking status during pregnancy, parity, and mode of delivery) stratified by Small of Gestational Age (SGA) for the 6 nominal statistically significant birthweight related metabolites and the 8 associated with overweight/obesity in early childhood. Where * is P< 0.05 and ** is FDR<0.05. Bars show 95% confidence intervals.

## ****Study population****

The ENVIRONAGE cohort recruits since 2010, and the sampling of this specific study population occurred between 2014 and 2015 in Belgium. Women were recruited when they arrived at the South-East-Limburg Hospital in Gent Follow-up anthropometric data collection for children is available up to two years of age. ENVIRONAGE study was approved by the ethical committees of Hasselt University and Hospital East-Limburg, Genk, Belgium. The INMA cohort is a network of birth cohorts in Spain that recruited pregnant women from the first trimester at public primary health care centers or hospitals in Sabadell from July 2004 to July 2006. Follow-up anthropometric data measurements, samples and surveys of the participating children have been collected until 16 years of age. INMA study was approved by the Ethical Committee of the Municipal Institute of Medical Investigation. The Piccolipiu study recruited women giving birth between 2011 and 2013 at selected hospitals in five Italian cities, Turin, Trieste, Viareggio, Florence, Rome. Children included in STOP were selected from the Turin center. Follow-up anthropometric data collection surveys occurred at 6, 12 and 24 months after the delivery and then when the children turned 4 and 6 years with direct measurements at a clinical visit. For Piccolipiu study, Ethical approvals have been obtained from the Ethics Committees of the Local Health Unit Roma E (management center), of the Istituto Superiore di Sanità (National Institute of Public Health), and of each local center. The Rhea cohort enrolled women during the first trimester of pregnancy at public primary health care centres or hospitals in Heraklion, Greece, between 2007 and 2008. Follow-up anthropometric measurements, samples and surveys for the participants are available up to 11 years. Follow-up anthropometric measurements, samples and surveys for the participants are available up to 11 years. Rhea was approved by the ethical committee of the University Hospital in Heraklion, Crete, Greece. For all studies, informed consent was given by all participants.

Venipuncture was used for collecting blood samples of cord vessels before the placenta was delivered. Samples were processed into either plasma (Environage, Piccolipiu) or serum (Rhea, INMA) as previously described^1^. Cohort inclusion criteria and further protocols can be found in the respective cohort references. Samples were selected from each cohort on the basis of biomaterial and data availability^2-5^. Selected samples were shipped to the International Agency for Research on Cancer, Lyon, France for metabolomics analysis.

Family lifestyle factors were collected from mothers through an interview by trained fieldworkers and medical history for each family transferred from hospital records^1^.

Regarding maternal diet during pregnancy (Table S1-S2), in the INMA cohort, an adapted version of Willett’s questionnaire^6^ was developed and validated for the Spanish population^7^.A Food Frequency Questionnaire (FFQ) was administered by trained interviewers during the 3rd trimester. The questionnaire consisted of question related to the frequency that a participant had consumed specific types of food^8^.The questionnaire had nine possible intake food categories, ranging from ‘never or less than once per month’ to ‘6 or more times per day’. The average daily food consumption calculated based on the overall intake frequency for each food item intake for each participant. In the RHEA cohort was developed a semi-quantitative questionnaire, containing 250 food items^8^. The participants were asked about both the frequency of consumption and the average portion size. The exact frequency of consumption was given per day, per week and/or per month, depending on the food item. The intake frequency for each food item was converted to the average daily intake for each participant. In the ENVIRONAGE cohort information on the maternal diet during the pregnancy was derived from the questionnaire filled out after delivery, including questions on the consumption of soft drinks, fish, fruit, and vegetable intake. Participants were asked for the frequency of average portion consumption per day and/or per week, depending on the food item. In the Piccolipiu cohort an FFQ for 13 items based on other questionnaires, but not ad hoc validated, was used.

## Untargeted metabolomics

Cord blood samples were prepared by protein precipitation and analyzed in randomized order as a single uninterrupted batch with a UHPLC-QTOF-MS system consisting of a 1290 Binary LC, a Jet Stream electrospray ionization source, and a 6550 QTOF mass spectrometer (Agilent Technologies). Details of the analysis have been described earlier Robinson, Keski-Rahkonen *et al* ^1^. In short, 30 μL of the sample was mixed with 200 μL of acetonitrile and filtered with 0.2 μm polypropene well plate filters, and the analysis was performed on a reversed phase column using a 13-minute methanol-water gradient. The mass spectrometer was operated in positive polarity with a mass range of 50-1000 Da. Preprocessing of the acquired data was carried out using Agilent’s recursive feature finding workflow as described earlier in detail ^1^. Briefly, a molecular feature extraction algorithm was used to find singly charged proton adducts, which were filtered by detection frequency and peak size into a target list of features, which were extracted from the raw data using a find-by-ion algorithm with a matching tolerance for the mass and retention time at ±10 ppm and ±0.04 min. Peak areas were used as a measurement of feature intensity. Metabolic features present in <60% of the samples were removed and data were log-transformed. Missing values were imputed leaving 4714 features for analysis using imputeLCMD R package ^9^. For identification of the features discovered in the present study, mass-to-charge ratios (m/z) were searched in the Human Metabolome Database ^10^ and METLIN ^11^, using ions [M+H]+, [M-H2O+H]+ and [M+Na]+, with 15 ppm molecular weight tolerance. Identity of the candidate metabolites was confirmed by reanalysis of representative samples together with pure chemical standards and comparing retention times and MS/MS spectra. When standards were not available, MS/MS spectra were acquired when possible and compared against those in public databases ([www.mzcloud.org](http://www.mzcloud.org), METLIN). Level of identification was defined as proposed by Sumner, Amberg *et al* ^12^. Chromatograms and mass spectra of all identified compounds are provided in the Supporting Information.

## Random Forest and model evaluation for optimism

A bootstrap method of 1000 repetitions was advocated to quantify optimism and evaluate the generalization of the model. In this analysis, we had two dependent variables to examine. The first dependent variable was the rapid growth at twelve months of age and the second in early childhood as it was defined in the main text. We used three different sets of independent variables for each of the outcomes: 1) traditional risk factors (cohort, ethnicity, maternal BMI, paternal BMI, gestational age, maternal weight gained during pregnancy, paternal education, maternal passive and active smoking status during pregnancy, parity and mode of delivery), 2)significantly associated metabolites from the MWAS analysis, and 3) significantly associated metabolites in combination with traditional risk factors. Al the models were adjusted for age and gender. A Random Forest classification model of 250 trees was trained on the relevant training set using Scikit-learn default parameters ^13^.

For all the bootstrapped models, we use a training set (random 80% of the total observations) to determine the optimum probability threshold, and the performance was evaluated on the relevant test set (remaining 20% of the total observations) for the cohorts that remained to the sample. The performance of all the models was assessed through receiver operating characteristic (ROC curve), and we estimate the bootstrapped 95% confidence intervals.

To further evaluate the predictive model, we performed a leave‐one‐out analysis by repeating the modelling process on a combined data set with one cohort out. We carried out this evaluation step following the above-mentioned methodology.

The results showed that the rapid growth prediction model trained using only traditional risk factors and exhibited a moderate predictive ability of an AUROC value of 0.69 (bootstrap 95% confidence interval (CI): 0.62, 0.77)). Adding the four metabolites (cholestenone, U2, U4, and U8) identified in the MWAS analysis into the prediction model, increased the AUROC to 0.77 (bootstrap 95% confidence interval (CI): 0.71, 0.83)) (Table S8). For overweight, using traditional risk factors alone, the AUROC was 0.69 (bootstrap 95% confidence interval (CI): 0.63, 0.75)), while a model using only the eight metabolites, Valine, U1, U2, U3, U4, U5, U7 and U9, identified in the MWAS analysis had an AUROC of 0.76 (bootstrap 95% confidence interval (CI): 0.69, 0.81)). The combined traditional risk factor and metabolite model was strongly predictive of overweight with an AUROC of 0.82 (bootstrap 95% confidence interval (CI): 0.79, 0.85)) (Table S8).

Table S8: Summary of rapid growth and 12 months of age and overweight/obesity in childhood. Average AUROC across 1000 bootstrapped test sets for all the cohorts.

| Model* | | Rapid growth at 12 months of age | | | Overweight/obesity in early childhood | | |
| --- | --- | --- | --- | --- | --- | --- | --- |
|  |  | Average AUROC | Lower 95%CI | Upper 95%CI | Average AUROC | Lower 95%CI | Upper 95%CI |
| 1 | Questionnaires** | 0.69 | 0.62 | 0.77 | 0.69 | 0.63 | 0.75 |
| 2 | Metabolomics*** | 0.72 | 0.64 | 0.81 | 0.76 | 0.69 | 0.81 |
| 3 | Metabolomics and questionnaires**** | 0.77 | 0.71 | 0.83 | 0.82 | 0.79 | 0.85 |

*All the models were adjusted for age and sex.

**Multivariate analysis for cohort, ethnicity, maternal BMI, paternal BMI, gestational age, maternal weight gained during pregnancy, paternal education, maternal passive and active smoking status during pregnancy, parity, and mode of delivery.

***Multivariate analysis of rapid growth at 12 months of age for Cholestenone, U4, U6 and U8 and of overweight/obesity in early childhood for Valine, U1, U2, U3, U4, U5, U7 and U9**** Multivariate model using the covariates of model 1 and 2.

Table S9: Summary of rapid growth and 12 months of age and overweight/obesity in childhood. Average ROC and CI95% across 1000 bootstrapped test sets using and leave-cohort-out approach.

| Model* | | Validation cohort | Rapid growth at 12 months of age | | | Overweight/obesity in early childhood | | |
| --- | --- | --- | --- | --- | --- | --- | --- | --- |
|  |  |  | Average AUROC | Lower 95%CI | Upper 95%CI | Average AUROC | Lower 95%CI | Upper 95%CI |
| 1 | Questionnaires** | ENVIRONAGE | 0.72 | 0.68 | 0.74 | - | - | - |
|  |  | Piccolipiu | 0.74 | 0.69 | 0.79 | 0.79 | 0.75 | 0.83 |
|  |  | RHEA | 0.61 | 0.57 | 0.66 | 0.63 | 0.61 | 0.65 |
|  |  | INMA-Sabadell | 0.80 | 0.77 | 0.83 | 0.68 | 0.64 | 0.72 |
| 2 | Metabolomics*** | ENVIRONAGE | 0.64 | 0.61 | 0.67 | - | - | - |
|  |  | Piccolipiu | 0.68 | 0.64 | 0.72 | 0.62 | 0.59 | 0.73 |
|  |  | RHEA | 0.74 | 0.73 | 0.75 | 0.75 | 0.71 | 0.78 |
|  |  | INMA-Sabadell | 0.70 | 0.67 | 0.73 | 0.62 | 0.58 | 0.64 |
| 3 | Metabolomics and questionnaires**** | ENVIRONAGE | 0.70 | 0.67 | 0.74 | - | - | - |
|  |  | Piccolipiu | 0.81 | 0.76 | 0.83 | 0.64 | 0.60 | 0.68 |
|  |  | RHEA | 0.65 | 0.61 | 0.68 | 0.79 | 0.74 | 0.83 |
|  |  | INMA-Sabadell | 0.82 | 0.79 | 0.85 | 0.71 | 0.68 | 0.74 |

*All the models were adjusted for age and sex.

**Multivariate analysis for ethnicity, maternal BMI, paternal BM, gestational age, maternal weight gained during pregnancy, paternal education, maternal passive and active smoking status during pregnancy, parity, and mode of delivery.

***Multivariate analysis of rapid growth at 12 months of age for Cholestenone, U4, U6 and U8 and of overweight/obesity in early childhood for Valine, U1, U2, U3, U4, U5, U7 and U9

****Multivariate model using the covariates of model 1 and 2.

The results of the leave one cohort out analysis (Table S9) for rapid growth showed an improvement in predictive performance upon addition of metabolites for Piccolipiu, Rhea and INMA as validation cohorts. For overweight, the leave one cohort out analysis (Table S9) showed an improvement in predictive performance upon addition of metabolites for Rhea and INMA as validation cohorts. These differences in predictive performance across cohorts may reflect the heterogeneity of the metabolic profiles we observed in each cohort and also, for overweight models, the lower age range and proportion of overweight cases in the Piccolipiu cohort (Figure S10).

The statistical analyses were performed using R (‘The R Project for Statistical Computing’) software environment (v3.5.2) and Python 3.6.


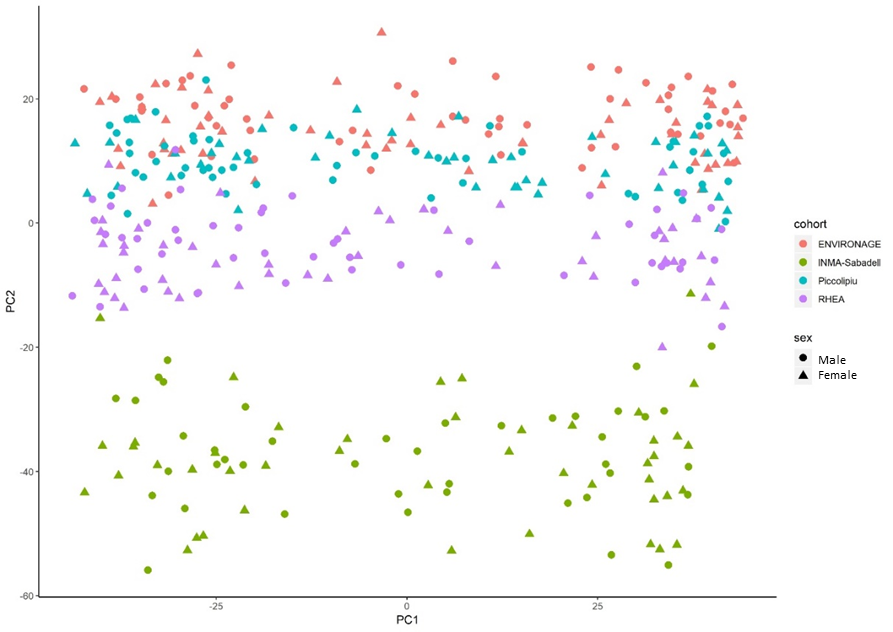


Figure S10: PCA analysis of the whole metabolome and scatter plot of first two principal components, coloured by cohort.

## Metabolic pathway enrichment analysis

We performed pathway enrichment analysis using Mummichog (version: 2.3.3-20200213, default metabolic human model MFN_1.10.4.). Mummichog is a bioinformatics Python-based platform that infers and categorizes functional biological activity using directly the output from mass spectrometry ^14^. The algorithm searches tentative compound lists from metabolite reference databases against an integrated model of human metabolism to identify functional activity. Fisher’s exact tests are used to infer p-values, which are adjusted for type I error through a pathway permutation procedure. Likelihood of pathway enrichment across significant features is compared to pathways identified across the entire compound set in a reference list (the entire metabolome dataset), considering the probability of mapping the significant metabolic features to pathways. Mummichog parameters were set to match against ions included in the ‘positive mode’ setting at ± 8 ppm mass tolerance ("M+H[1+]" and "M+Na[1+]").

Mummichog assigned tentative annotations to 405 of the 4714 features as significant (P<0.05) for rapid growth in 12 months (Supporting information 2) and to 613 of the 4714 for overweight/obesity in early childhood (Supporting information 3). Mummichog reference feature list was mapped to 627 Empirical Compounds which 69 were statistically significant for rapid growth in 12 months and 78 statistically significant for overweight/obesity in early childhood. According Mummichog, Empirical Compounds are putative metabolites as measured by Liquid chromatography coupled to high-resolution mass spectrometry (LC-HRMS). These putative metabolites can contain a mixture of enantiomers, stereoisomers, and positional isomers that are not resolved by the instruments^15^.

The results showed that the three enriched pathways with overlap size ≥4 for rapid growth in infancy were “Androgen and estrogen biosynthesis and metabolism”, “C21-steroid hormone biosynthesis and metabolism” and “Urea cycle/amino group metabolism” (Table S10) and enriched pathways with overlap size ≥4 for overweight/obesity in early childhood were “Valine, leucine and isoleucine degradation”, “Biopterin metabolism” and “Glycine, serine, alanine and threonine metabolism”(Table S11).

Additionally, to further validate the pathways proposed by mummichog, we carried out a manual curation of the metabolite identities assigned by mummichog. For the compounds previously identified by the laboratory and for which pure chemical standards were available, retention times were compared to exclude false mummichog annotations.

The results of this manual validation lend support for the correctness of following mummichog-predicted pathways: for rapid growth, “C21-steroid hormone biosynthesis and metabolism”, “androgen and estrogen biosynthesis and metabolism”, and “Urea cycle/amino group metabolism” retained overlap sizes of 13,12 and 5, respectively after excluding the false metabolite annotations (Table S10). For overweight, “Glycine, serine, alanine and threonine metabolism” retained an overlap size of 5, although the statistical support was weak (p = 0.05 before manual exclusion) (Table S11).

Table S10: Mummichog analysis statistically significant pathways for rapid growth at 12 months of age.

| Pathways | Overlap size^A^ | Pathway size^B^ | p-value^C^ | Overlap empirical compounds^E^ |
| --- | --- | --- | --- | --- |
| C21-steroid hormone biosynthesis and metabolism | 15 | 58 | 8e-05 | E285, E479, E151^D^, E423, E487^D^, E124, E181, E309, E386, E539, E219, E382, E379, E416, E36 |
| Androgen and estrogen biosynthesis and metabolism | 12 | 30 | 8e-05 | E285, E386, E36, E423, E124, E416, E309, E219, E539, E463, E209, E382 |
| Urea cycle/amino group metabolism | 8 | 34 | 0.007 | E288^D^, E387, E94, E57^D^, E488^D^, E98, E548^D^, E37^D^ |

A Pathway size is number of detected Empirical Compounds for each pathway.

B Overlap size is number of significant Empirical Compounds.

C Empirical p-values are estimated by permutation test.

D This empirical compound has not been identified in the manual identification.

E Details on empirical compounds are available in supporting information 2.

Table S11: Mummichog analysis statistically significant pathways for overweight/obesity in early childhood.

| Pathways | Overlap size^A^ | Pathway size^B^ | p-value^C^ | Overlap empirical compounds^E^ |
| --- | --- | --- | --- | --- |
| Valine, leucine and isoleucine degradation | 6 | 13 | 0.0006 | E34, E17, E239, E350^D^, E549^D^, E180^D^ |
| Biopterin metabolism | 4 | 9 | 0.005 | E484^D^, E601, E290^D^, E175 |
| Glycine, serine, alanine and threonine metabolism | 8 | 42 | 0.050 | E17, E350^D^, E449, E3, E407, E549^D^, E180^D^, E394 |

A Pathway size is number of detected Empirical Compounds for each pathway.

B Overlap size is number of significant Empirical Compounds.

C Empirical p-values are estimated by permutation test.

D This empirical compound has not been identified in the manual identification.

E Details on empirical compounds are available in supporting information 3.

## Modelling of weight and height growth trajectories

Patterns of growth across childhood follow a complex pattern (growth is non-linear). We used a two-step approach to estimate growth curves for participating cohorts. First, we identified for each cohort the best fitting fractional polynomials of age and constructed sex-and age- specific weight and height growth curves^16^. Briefly, a series of models were carried out for each cohort in which age was raised to a large number of combinations of powers (each of the following single powers, plus each combination of two powers: −2, −1, −0.5, 0, 0.5, 1, 2, 3, where a power of zero is the log function), resulting in a wide range of possible weight and height curves^17^. Then we used mixed-effects linear regression models with the previously identified fractional polynomials of age, including a random intercept for child and random age slopes. Such models allow for individual variation in growth curves within each cohort, and use all available data from all the eligible children under a missing at random assumption^18^. Predicted weight and height values within each cohort were estimated for the exact age of 12months for the cohorts’ individuals.

We used the WHO growth charts to monitor child growth ^19, 20^. These charts are growth standards based on data collected from selected communities worldwide. The use of WHO standards allows for growth assessment of children independent of ethnicity and socioeconomic status, thus, permitting international comparisons. These charts have been adopted in a growing number of countries in Europe and other parts of the world ^21^, and endorsed by international bodies such as the United Nations Standing Committee on Nutrition ^22^ and International Pediatric Association ^23^.

The selected models are available in Table S12 and the performance of the models are presented in Figure S11-S14.

Table S12: Comparison of prediction concordance from different fractional polynomial powers for sex-specific weight and height in participating cohorts.

|  | | | **Boys** | | | | | | | | | | **Girls** | | | | | | | | | |
| --- | --- | --- | --- | --- | --- | --- | --- | --- | --- | --- | --- | --- | --- | --- | --- | --- | --- | --- | --- | --- | --- | --- |
|  |  |  | **Weight** | | | | | **Height** | | | | | **Weight** | | | | | **Height** | | | | |
| **Cohort** | **N** | **n** | **Powers** | | **rho_c_*** | **Difference** | | **Powers** | | **rho_c_*** | **Difference** | | **Powers** | | **rho_c_*** | **Difference** | | **Powers** | | **rho_c_*** | **Difference** | |
|  |  |  |  |  |  | **Mean** | **(SD)** |  |  |  | **Mean** | **(SD)** |  |  |  | **Mean** | **(SD)** |  |  |  | **Mean** | **(SD)** |
| ENVIRONAGE | 108 | 1104 | -2 | 0.5 | 0.994 | -0.001 | (0.477) | -2 | 0.5 | 0.998 | -0.011 | (1.134) | 0 | 0 | 0.996 | -0.001 | (0.408) | -1 | 0.5 | 0.998 | -0.006 | (1.048) |
| INMA-Sabadell | 404 | 3149 | 0.5 | 3 | 0.997 | -0.000 | (0.592) | 0 | 1 | 0.998 | -0.000 | (1.415) | 0.5 | 3 | 0.997 | 0.000 | (0.506) | 0.5 | 3 | 0.998 | -0.000 | (1.417) |
| PICCOLIPIU | 99 | 943 | 0 | 0 | 0.991 | -0.000 | (0.359) | 0 | 0 | 0.989 | -0.000 | (1.537) | 0.5 | 1 | 0.992 | 0.000 | (0.300) | 0 | 0 | 0.989 | -0.000 | (1.456) |
| RHEA | 1092 | 21045 | 0 | 0.5 | 0.989 | -0.000 | (0.858) | 0.5 | 0.5 | 0.997 | 0.000 | (1.530) | 0.5 | 3 | 0.996 | -0.000 | (0.502) | 0.5 | 1 | 0.997 | 0.000 | (1.384) |

* ***rho_c_***: concordance correlation coefficient.

Figure S11: Actual vs Predicted values of weight and height in participating ENVIRONAGE cohort.

Figure S12: Actual vs Predicted values of weight and height in participating INMA cohort.

Figure S13: Actual vs Predicted values of weight and height in participating Piccolipiu cohort.

Figure S14: Actual vs Predicted values of weight and height in participating RHEA cohort.

## References

1. Robinson O, Keski-Rahkonen P, Chatzi L, Kogevinas M, Nawrot T, Pizzi C *et al.* Cord blood metabolic signatures of birth weight: a population-based study. *Journal of proteome research* 2018; **17**(3)**:** 1235-1247.

2. Chatzi L, Leventakou V, Vafeiadi M, Koutra K, Roumeliotaki T, Chalkiadaki G *et al.* Cohort profile: the mother-child cohort in Crete, Greece (Rhea study). 2017; **46**(5)**:** 1392-1393k.

3. Janssen BG, Madhloum N, Gyselaers W, Bijnens E, Clemente DB, Cox B *et al.* Cohort profile: the ENVIRonmental influence ON early AGEing (ENVIR ON AGE): a birth cohort study. 2017; **46**(5)**:** 1386-1387m.

4. Farchi S, Forastiere F, Vecchi Brumatti L, Alviti S, Arnofi A, Bernardini T *et al.* Piccolipiù, a multicenter birth cohort in Italy: protocol of the study. *BMC pediatrics* 2014; **14:** 36-36.

5. Guxens M, Ballester F, Espada M, Fernández MF, Grimalt JO, Ibarluzea J *et al.* Cohort profile: the INMA—INfancia y Medio Ambiente—(environment and childhood) project. *International journal of epidemiology* 2011; **41**(4)**:** 930-940.

6. Willett WC, Sampson L, Stampfer MJ, Rosner B, Bain C, Witschi J *et al.* Reproducibility and validity of a semiquantitative food frequency questionnaire. *American journal of epidemiology* 1985; **122**(1)**:** 51-65.

7. Vioque J, Weinbrenner T, Asensio L, Castelló A, Young IS, Fletcher A. Plasma concentrations of carotenoids and vitamin C are better correlated with dietary intake in normal weight than overweight and obese elderly subjects. *British journal of nutrition* 2007; **97**(5)**:** 977-986.

8. Chatzi L, Mendez M, Garcia R, Roumeliotaki T, Ibarluzea J, Tardón A *et al.* Mediterranean diet adherence during pregnancy and fetal growth: INMA (Spain) and RHEA (Greece) mother–child cohort studies. *British Journal of Nutrition* 2012; **107**(1)**:** 135-145.

9. Lazar C. imputeLCMD: a collection of methods for left-censored missing data imputation. *R package, version* 2015; **2**.

10. Wishart DS, Feunang YD, Marcu A, Guo AC, Liang K, Vázquez-Fresno R *et al.* HMDB 4.0: the human metabolome database for 2018. *Nucleic Acids Res* 2018; **46**(D1)**:** D608-d617.

11. Smith CA, O'Maille G, Want EJ, Qin C, Trauger SA, Brandon TR *et al.* METLIN: a metabolite mass spectral database. *Ther Drug Monit* 2005; **27**(6)**:** 747-51.

12. Sumner LW, Amberg A, Barrett D, Beale MH, Beger R, Daykin CA *et al.* Proposed minimum reporting standards for chemical analysis Chemical Analysis Working Group (CAWG) Metabolomics Standards Initiative (MSI). *Metabolomics* 2007; **3**(3)**:** 211-221.

13. Pedregosa F, Varoquaux G, Gramfort A, Michel V, Thirion B, Grisel O *et al.* Scikit-learn: Machine learning in Python. *Journal of machine learning research* 2011; **12**(Oct)**:** 2825-2830.

14. Li S, Park Y, Duraisingham S, Strobel FH, Khan N, Soltow QA *et al.* Predicting network activity from high throughput metabolomics. *PLoS computational biology* 2013; **9**(7)**:** e1003123.

15. Pang Z, Chong J, Li S, Xia J. MetaboAnalystR 3.0: Toward an optimized workflow for global metabolomics. *Metabolites* 2020; **10**(5)**:** 186.

16. Royston P, Wright EM. A method for estimating age‐specific reference intervals (‘normal ranges’) based on fractional polynomials and exponential transformation. *Journal of the Royal Statistical Society: Series A (Statistics in Society)* 1998; **161**(1)**:** 79-101.

17. Long J, Ryoo J. Using fractional polynomials to model non‐linear trends in longitudinal data. *British Journal of Mathematical and Statistical Psychology* 2010; **63**(1)**:** 177-203.

18. Johnson W, Balakrishna N, Griffiths PL. Modeling physical growth using mixed effects models. *American journal of physical anthropology* 2013; **150**(1)**:** 58-67.

19. WHO Multicentre Growth Reference Study Group. *WHO Child Growth Standards: Length/height-for-age, weight-for-age, weight-for-length, weight-for-height and body mass index-for-age: Methods and development.*, World Health Organization: Geneva, 2006.

20. de Onis M, Onyango AW, Borghi E, Siyam A, Nishida C, Siekmann J. Development of a WHO growth reference for school-aged children and adolescents. *Bull World Health Organ* 2007; **85**(9)**:** 660-7.

21. Turck D, Michaelsen KF, Shamir R, Braegger C, Campoy C, Colomb V *et al.* World Health Organization 2006 child growth standards and 2007 growth reference charts: A discussion paper by the committee on Nutrition of the European Society for Pediatric Gastroenterology, Hepatology, and Nutrition. *J. Pediatr. Gastroenterol. Nutr.* 2013; **57**(2)**:** 258-64.

22. UN Standing Committee on Nutrition. UN Standing Committee on Nutrition (SCN) Endorses the New WHO Growth Standards for Infants and Young Children. . In.

23. International Pediatric Association Endorsement. International Pediatric Association Endorsement. The New WHO Growth Standards for Infants and Young Children. In.
