# Supplementary material for: Cord blood metabolic signatures predictive of childhood overweight and rapid growth
Source: Int J Obes (Lond). 2021 Jul 12;45(10):2252–60. doi: 10.1038/s41366-021-00888-1 (PMC8455328; doi:10.1038/s41366-021-00888-1)
Supplement: Supplementary file 2 — Supporting information 2 [file 41366_2021_888_MOESM2_ESM.zip › result.html]

Mummichog Report


# Mummichog Report: rg

mummichog version: 2.1.1-beta-20180623, using metabolic model MFN\_1.10.4.

## User input data

User supplied 4712 features as reference list.We are using 405 features (p < 0.050000) as significant list. The feature level data are shown in the Manhattan plots below.

## Top pathways

The reference feature list is mapped to 627 EmpiricalCompounds, significant features to 69 EmpiricalCompounds. In the table below, pathway\_size is number of detected EmpiricalCompounds for each pathway; overlap\_size is number of significant EmpiricalCompounds. Empirical p-values are estimated by permutation test. Details on EmpiricalCompounds are in 'tables/ListOfEmpiricalCompounds.tsv'.

| Pathways | overlap\_size | pathway\_size | p-value | overlap\_EmpiricalCompounds |
| --- | --- | --- | --- | --- |
| C21-steroid hormone biosynthesis and metabolism | 15 | 58 | 8e-05 | E285,E479,E151,E423,E487,E124,E181,E309,E386,E539,E219,E382,E379,E416,E36 |
| Androgen and estrogen biosynthesis and metabolism | 12 | 30 | 8e-05 | E285,E386,E36,E423,E124,E416,E309,E219,E539,E463,E209,E382 |
| Urea cycle/amino group metabolism | 8 | 34 | 0.00681 | E288,E387,E94,E57,E488,E98,E548,E37 |
| Vitamin D3 (cholecalciferol) metabolism | 3 | 7 | 0.00941 | E47,E196,E253 |
| Drug metabolism - other enzymes | 1 | 1 | 0.04605 | E441 |
| Prostaglandin formation from dihomo gama-linoleic acid | 1 | 1 | 0.04605 | E52 |
| Vitamin D | 1 | 1 | 0.04605 | E253 |

## Top modules

Network modules may capture concerted metabolite activities that are missed by predefined pathways. Use pull-down menus to visualize modules and the activity network, which tries to combine the results of pathway/module analyses. The visualization requires interenet connection.

Showing
Module 1Module 2Module 3 Activity network
Node size 
Medium
Small
Large
Visual style 
Drag
Force

module\_1, p=0.00098, 31 metabolites

3alpha-Hydroxy-5beta-androstan-17-one; Etiocholan-3alpha-ol-17-one; 3alpha-Hydroxyetiocholan-17-one, Androstenedione, Androst-4-ene-3,17-dione; Androstenedione; 4-Androstene-3,17-dione, 17alpha,21-Dihydroxy-5beta-pregnane-3,11,20-trione; 5beta-Pregnane-17alpha,21-diol-3,11,20-trione; 4,5beta-Dihydrocortisone, 5alpha-Dihydrotestosterone, , 18-Hydroxycorticosterone, 3beta-Hydroxyandrost-5-en-17-one; Dehydroepiandrosterone; Dehydroisoandrosterone; DHA; DHEA, 11-Deoxycortisol; Cortodoxone, Progesterone; 4-Pregnene-3,20-dione, Testosterone, Androst-5-ene-3beta,17beta-diol; 3beta,17beta-Dihydroxyandrost-5-ene; 3beta,17beta-Dihydroxy-5-androstene; Androstenediol, 19-Hydroxytestosterone; 17beta,19-Dihydroxyandrost-4-en-3-one, 5beta-Dihydrotestosterone, Corticosterone; 11beta,21-Dihydroxy-4-pregnene-3,20-dione; Kendall's compound B; Reichstein's substance H, 6beta-Hydroxytestosterone; 6beta,17beta-Dihydroxyandrost-4-en-3-one, Cholest-4-en-3-one; Cholestenone; 4-Cholesten-3-one, 11beta,21-Dihydroxy-3,20-oxo-5beta-pregnan-18-al, 2beta-hydroxytestosterone, Pregnenolone; 5-Pregnen-3beta-ol-20-one; 3beta-Hydroxypregn-5-en-20-one, 21-Hydroxy-5beta-pregnane-3,11,20-trione, 5beta-Pregnane-3,20-dione, 11beta-hydroxytestosterone, 5alpha-androstane-3alpha,17beta-diol, 21-Deoxycortisol; 4-Pregnene-11beta,17alpha-diol-3,20-dione; 11beta,17alpha-Dihydroxyprogesterone, 16alpha-Hydroxydehydroepiandrosterone; 5-Androstene-3beta,16alpha-diol-17-one, 5beta-Androstane-3,17-dione, 20alpha-Hydroxy-4-pregnen-3-one; 20alpha-Hydroxypregn-4-en-3-one; 20alpha-Hydroxyprogesterone, Androsterone, Cortisol; Hydrocortisone; 11beta,17alpha,21-Trihydroxy-4-pregnene-3,20-dione; Kendall's compound F; Reichstein's substance M, 5alpha-Androstane-3,17-dione; Androstanedione

module\_2, p=0.00098, 11 metabolites

Cholest-4-en-3-one; Cholestenone; 4-Cholesten-3-one, 3alpha-Hydroxy-5beta-androstan-17-one; Etiocholan-3alpha-ol-17-one; 3alpha-Hydroxyetiocholan-17-one, 21-Hydroxy-5beta-pregnane-3,11,20-trione, 5beta-Pregnane-3,20-dione, 5beta-Androstane-3,17-dione, 17alpha,21-Dihydroxy-5beta-pregnane-3,11,20-trione; 5beta-Pregnane-17alpha,21-diol-3,11,20-trione; 4,5beta-Dihydrocortisone, Progesterone; 4-Pregnene-3,20-dione, 11beta,21-Dihydroxy-3,20-oxo-5beta-pregnan-18-al, 20alpha-Hydroxy-4-pregnen-3-one; 20alpha-Hydroxypregn-4-en-3-one; 20alpha-Hydroxyprogesterone, 5beta-Dihydrotestosterone, Corticosterone; 11beta,21-Dihydroxy-4-pregnene-3,20-dione; Kendall's compound B; Reichstein's substance H

module\_3, p=0.00098, 10 metabolites

3beta-Hydroxyandrost-5-en-17-one; Dehydroepiandrosterone; Dehydroisoandrosterone; DHA; DHEA, Androst-4-ene-3,17-dione; Androstenedione; 4-Androstene-3,17-dione, 11-Deoxycortisol; Cortodoxone, 16alpha-Hydroxydehydroepiandrosterone; 5-Androstene-3beta,16alpha-diol-17-one, Androst-5-ene-3beta,17beta-diol; 3beta,17beta-Dihydroxyandrost-5-ene; 3beta,17beta-Dihydroxy-5-androstene; Androstenediol, Cortisol; Hydrocortisone; 11beta,17alpha,21-Trihydroxy-4-pregnene-3,20-dione; Kendall's compound F; Reichstein's substance M, 21-Deoxycortisol; 4-Pregnene-11beta,17alpha-diol-3,20-dione; 11beta,17alpha-Dihydroxyprogesterone, Pregnenolone; 5-Pregnen-3beta-ol-20-one; 3beta-Hydroxypregn-5-en-20-one, , 18-Hydroxycorticosterone

## EmpiricalCompounds favored by above analyses

An EmpiricalCompound is a computational unit for a tentative metabolite. It can group multiple ions, and be any of the isobaric/isomeric species. This table contains EmpiricalCompounds prioritized by pathway/module analysis.

| EmpiricalCompound | Input m/z | Retention time | ion | mz\_diff | Statistic | Significant |
| --- | --- | --- | --- | --- | --- | --- |
| E387 | Best guess: C00408, L-Pipecolate | | C05936, C00408 | | | |
|  | 130.08638 | 3.4959247 | M+H[1+] | 0.0001 | -2.03 | yes |
| E196 | Best guess: CE2204, 25-hydroxyvitamin D3-26,23-lactone | | CE2204 | | | |
|  | 429.29858 | 6.101364 | M+H[1+] | -0.0014 | 2.99 | yes |
| E479 | Best guess: C00410, Progesterone | | C00410 | | | |
|  | 337.21388 | 6.394628 | M+Na[1+] | 0.0 | 2.71 | yes |
|  | 315.23198 | 6.3944817 | M+H[1+] | 0.0001 | 2.65 | yes |
| E309 | Best guess: C11133, Estrone glucuronide | | estroneglc, C11133 | | | |
|  | 447.20148 | 4.453114 | M+H[1+] | 0.0001 | -3.23 | yes |
| E386 | Best guess: C05293, 5beta-Dihydrotestosterone | | C04373, C03917, C00523, C04295, C05293 | | | |
|  | 313.21588 | 4.8733563 | M+Na[1+] | 0.002 | -3.02 | yes |
| E463 | Best guess: CE2209, 5alpha-androstane-3alpha,17beta-diol | | CE2209 | | | |
|  | 315.23198 | 6.3944817 | M+Na[1+] | 0.0025 | 2.65 | yes |
| E37 | Best guess: C01110, 5-Amino-2-oxopentanoic acid | | C00430, C03440, C01165, C03341, C01157, C01110 | | | |
|  | 132.06658 | 0.8810777 | M+H[1+] | 0.0011 | 2.12 | yes |
|  | 154.04778 | 0.87768334 | M+Na[1+] | 0.0003 | 1.58 | no |
| E36 | Best guess: C05504, 16-Glucuronide-estriol | | C05504 | | | |
|  | 487.19398 | 4.4549704 | M+Na[1+] | 0.0001 | -2.96 | yes |
| E288 | Best guess: C00101, 5,6,7,8-Tetrahydrofolate | | C00101 | | | |
|  | 446.17828 | 6.3946886 | M+H[1+] | 0.0 | 2.51 | yes |
| E151 | Best guess: C00410, Progesterone | | C00410 | | | |
|  | 337.21468 | 5.722147 | M+Na[1+] | 0.0008 | -2.89 | yes |
|  | 315.23128 | 5.7192435 | M+H[1+] | -0.0006 | -2.82 | yes |
| E124 | Best guess: andrstndn, Androstenedione | | C00280, andrstndn | | | |
|  | 287.20048 | 5.309032 | M+H[1+] | -0.0001 | -3.55 | yes |
|  | 309.18378 | 5.3093767 | M+Na[1+] | 0.0012 | -3.45 | yes |
| E98 | Best guess: C01110, 5-Amino-2-oxopentanoic acid | | C00430, C03440, C01165, C03341, C01157, C01110 | | | |
|  | 132.06548 | 1.450143 | M+H[1+] | 0.0 | 2.09 | yes |
| E57 | Best guess: C01250, N-Acetyl-L-glutamate 5-semialdehyde | | C01250 | | | |
|  | 196.05998 | 2.9204202 | M+Na[1+] | 0.0019 | 3.58 | yes |
| E548 | Best guess: C01586, Hippurate | | CE5536, C01586 | | | |
|  | 180.06688 | 3.0666792 | M+H[1+] | 0.0014 | -2.65 | yes |
|  | 202.04828 | 3.0665774 | M+Na[1+] | 0.0008 | -2.87 | yes |
| E219 | Best guess: C01227, 3beta-Hydroxyandrost-5-en-17-one | | C00535, C03772, C00674, dhea, C01227 | | | |
|  | 289.21568 | 4.8316393 | M+H[1+] | -0.0005 | -4.5 | yes |
| E423 | Best guess: C05479, 5beta-Pregnane-3,20-dione | | C03681, C01953, C04042, C05479 | | | |
|  | 339.22878 | 5.731802 | M+Na[1+] | -0.0007 | -2.15 | yes |
|  | 317.24778 | 5.728532 | M+H[1+] | 0.0003 | -2.08 | yes |
| E487 | Best guess: C01921, glycocholate | | chsterols, C01921 | | | |
|  | 466.31408 | 5.897524 | M+H[1+] | -0.0022 | 2.64 | yes |
| E94 | Best guess: C05145, 3-Aminoisobutyric acid | | C01026, C01205, C02356, C00334, C03284, C05145 | | | |
|  | 104.07088 | 3.1786304 | M+H[1+] | 0.0003 | -2.09 | yes |
| E181 | Best guess: C01124, 18-Hydroxycorticosterone | | C00735, C05469, C05473, C01124 | | | |
|  | 363.21698 | 5.1705174 | M+H[1+] | 0.0004 | -3.11 | yes |
| E382 | Best guess: C05141, Estriol | | C05301, CE2179, hestratriol, C05141 | | | |
|  | 311.16108 | 4.699778 | M+Na[1+] | -0.0007 | 2.19 | yes |
| E285 | Best guess: C05139, 16alpha-Hydroxydehydroepiandrosterone | | CE1356, CE1358, C05294, C18075, C18045, C14497, C05139 | | | |
|  | 327.19388 | 5.2929516 | M+Na[1+] | 0.0008 | -2.6 | yes |
| E441 | Best guess: C16633, 5-Fluorouridine | | C16633 | | | |
|  | 263.06908 | 5.4294977 | M+H[1+] | 0.0017 | 2.56 | yes |
| E379 | Best guess: C05497, 21-Deoxycortisol | | CE1347, C02140, C05488, C05477, C05497 | | | |
|  | 347.22178 | 5.531753 | M+H[1+] | 0.0001 | -2.09 | yes |
| E47 | Best guess: CE2202, 23S,25,26-trihydroxyvitamin D3 | | C17335, CE2205, CE2202 | | | |
|  | 455.31238 | 6.3865395 | M+Na[1+] | -0.0009 | -0.9 | no |
|  | 455.31168 | 6.4861465 | M+Na[1+] | -0.0016 | -2.28 | yes |
| E253 | Best guess: C05443, Vitamin D3 | | C01802, C00599, C01164, C05439, C05437, C05443 | | | |
|  | 385.34868 | 9.076708 | M+H[1+] | 0.0022 | 5.49 | yes |
|  | 407.32988 | 9.073516 | M+Na[1+] | 0.0014 | 5.08 | yes |
| E416 | Best guess: C05479, 5beta-Pregnane-3,20-dione | | C03681, C01953, C04042, C05479 | | | |
|  | 339.23048 | 6.348832 | M+Na[1+] | 0.001 | 2.56 | yes |
|  | 317.24718 | 6.3491964 | M+H[1+] | -0.0003 | 2.03 | yes |
| E488 | Best guess: C00437, N2-Acetyl-L-ornithine | | C00437 | | | |
|  | 175.10788 | 0.6756697 | M+H[1+] | 0.0002 | -3.75 | yes |
| E539 | Best guess: andrstndn, Androstenedione | | C00280, andrstndn | | | |
|  | 287.20118 | 4.8652577 | M+H[1+] | 0.0006 | -2.79 | yes |
| E52 | Best guess: CE5828, | | CE6234, CE5924, CE5929, CE4989, CE5931, CE7093, CE5663, C08012, CE5828 | | | |
|  | 376.22448 | 7.6913433 | M+Na[1+] | 0.0024 | 2.23 | yes |
| E209 | Best guess: CE2209, 5alpha-androstane-3alpha,17beta-diol | | CE2209 | | | |
|  | 315.23128 | 5.7192435 | M+Na[1+] | 0.0018 | -2.82 | yes |

## More data

Full data tables from pathway analysis, module analysis and activity network are stored in the 'tables/' directory.
The tsv files can be imported into a spreadsheet program, e.g. MS Excel.
Under the 'figures/network\_modules/' directory are files intended for Cytoscape (cytoscape.org) visualization. Please refer to Cytoscape's guides for details.
Details of this run are recorded in file mummichog.log.txt.

Mummichog algorithms are described in Li et al. Predicting Network Activity from High Throughput Metabolomics. PLoS Computational Biology (2013); doi:10.1371/journal.pcbi.1003123. This software is provided as is, without warranty of any kind. Visit http://mummichog.org for updates.
  
