# Supplementary figures and images for: Cord blood metabolic signatures predictive of childhood overweight and rapid growth
Source: Int J Obes (Lond). 2021 Jul 12;45(10):2252–60. doi: 10.1038/s41366-021-00888-1 (PMC8455328; doi:10.1038/s41366-021-00888-1)

Modeling module significance

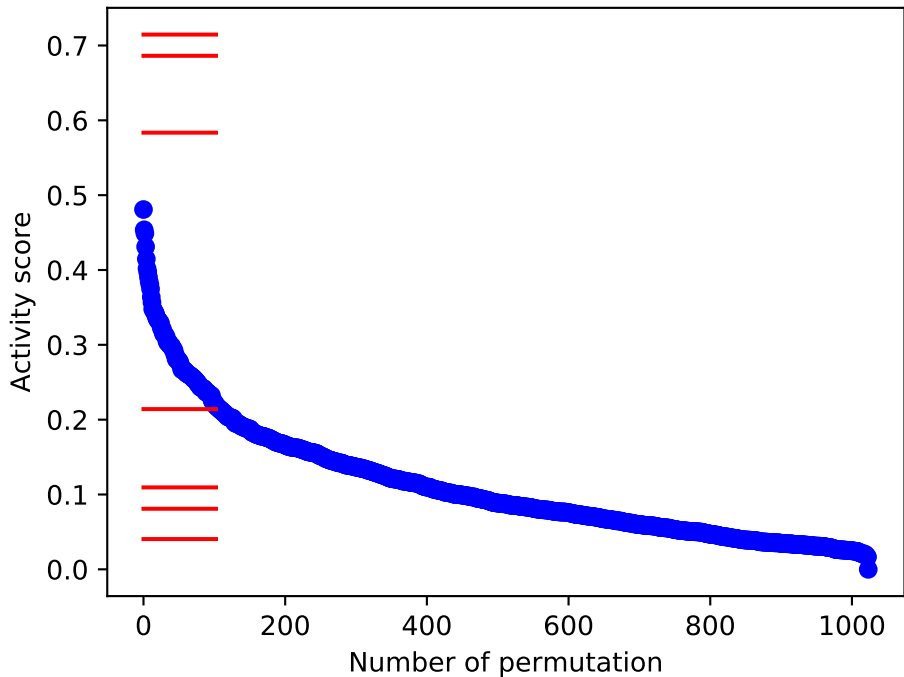

Supplement: Supplementary file 2 — Supporting information 2 [file 41366_2021_888_MOESM2_ESM.zip › figures/plot_moduleModel_rg.pdf]

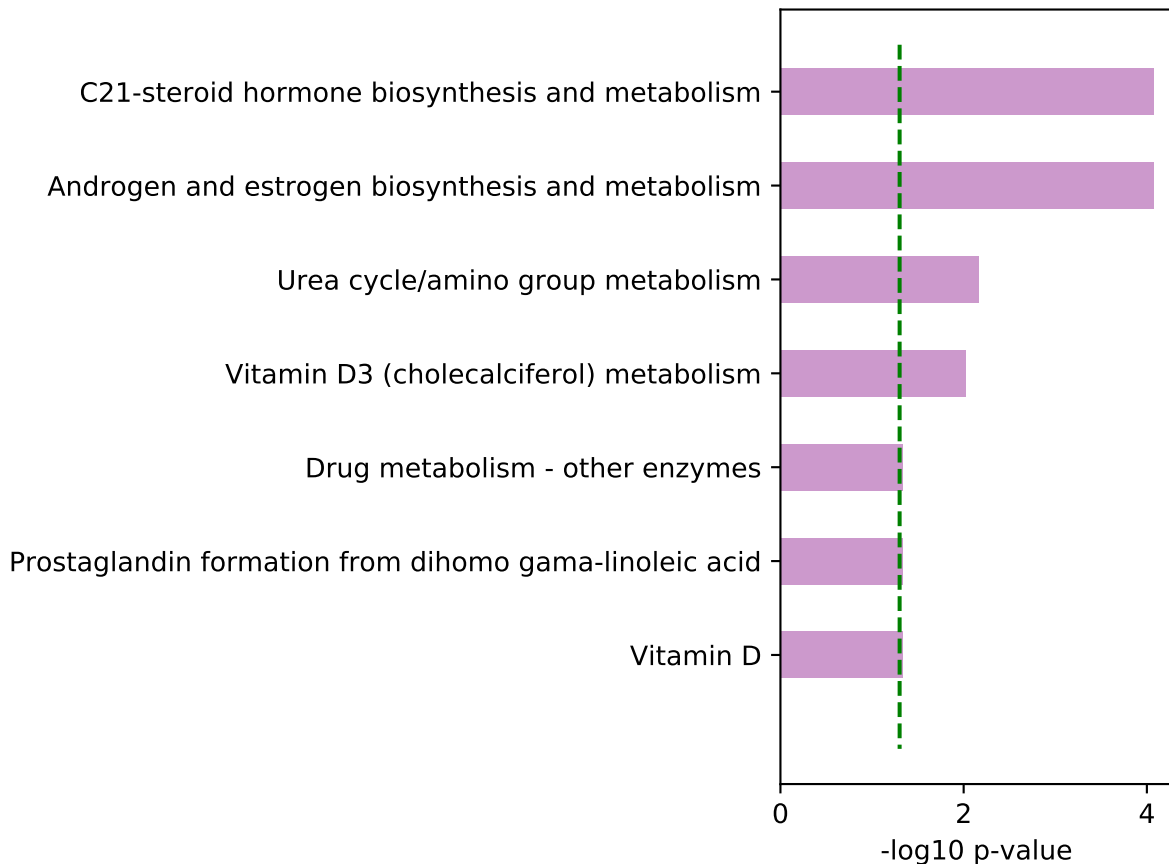

Supplement: Supplementary file 2 — Supporting information 2 [file 41366_2021_888_MOESM2_ESM.zip › figures/mcg_pathwayBars_rg.pdf]

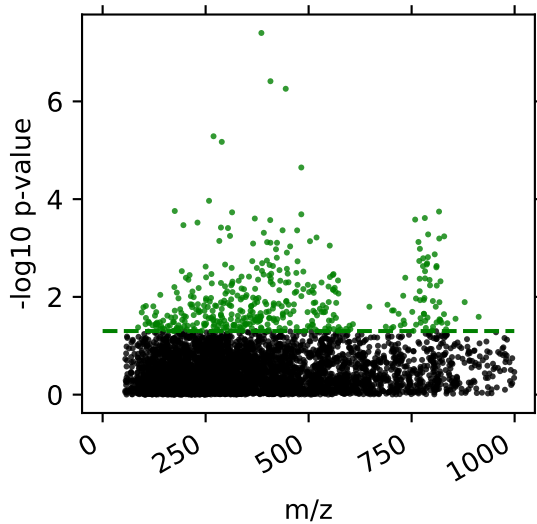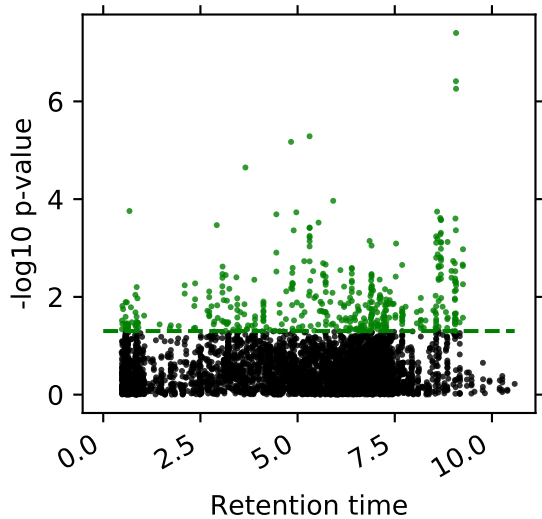

Supplement: Supplementary file 2 — Supporting information 2 [file 41366_2021_888_MOESM2_ESM.zip › figures/mcg_MWAS_rg.pdf]

## Modeling pathway significance

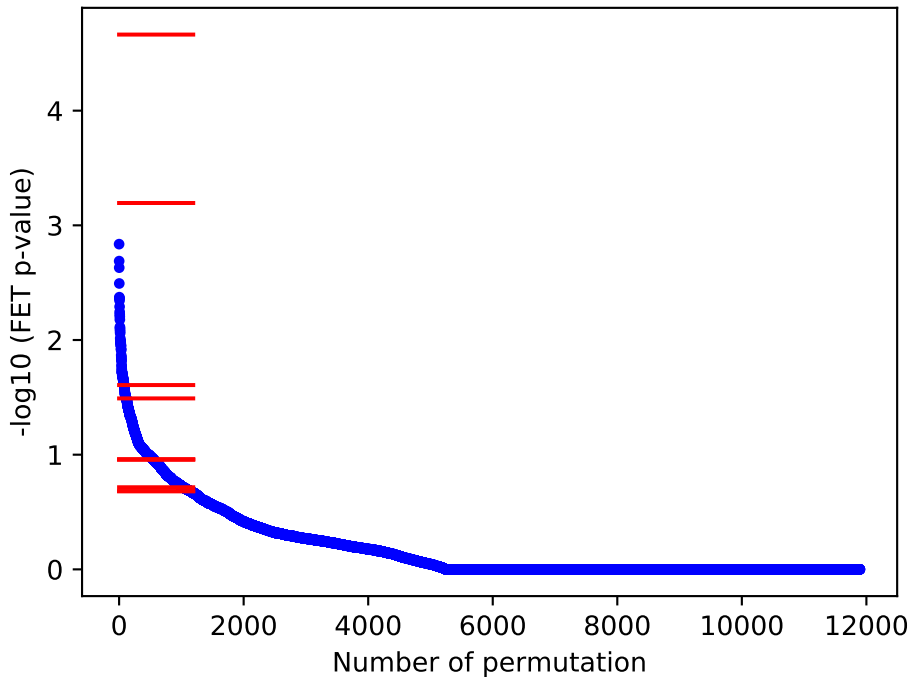

Supplement: Supplementary file 2 — Supporting information 2 [file 41366_2021_888_MOESM2_ESM.zip › figures/plot_pathwayModel_rg.pdf]

Modeling module significance

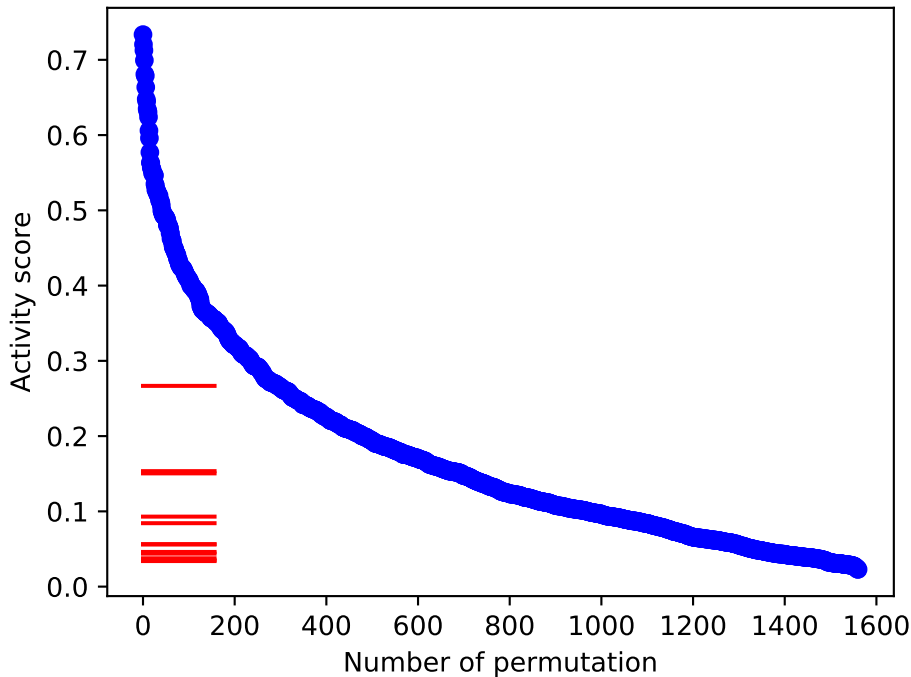

Supplement: Supplementary file 3 — Supporting information 3 [file 41366_2021_888_MOESM3_ESM.zip › figures/plot_moduleModel_.pdf]

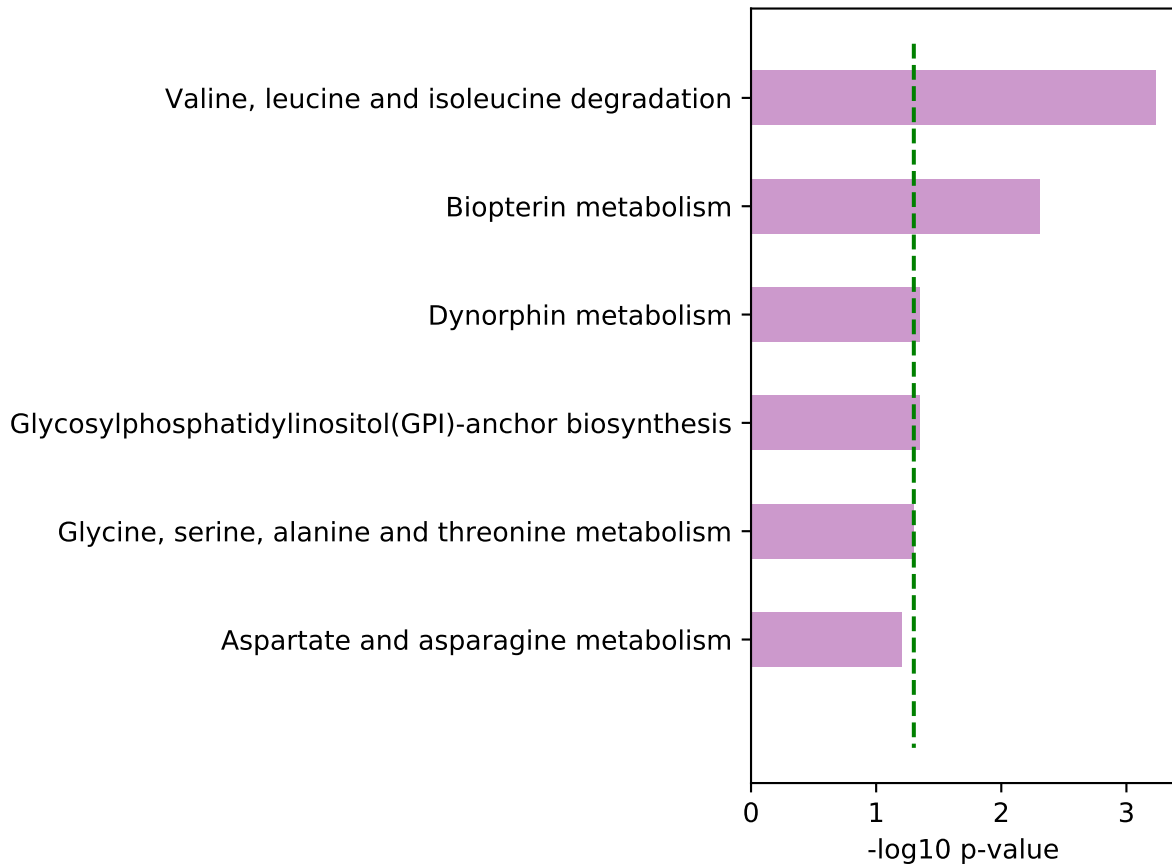

Supplement: Supplementary file 3 — Supporting information 3 [file 41366_2021_888_MOESM3_ESM.zip › figures/mcg_pathwayBars_.pdf]

# Modeling pathway significance

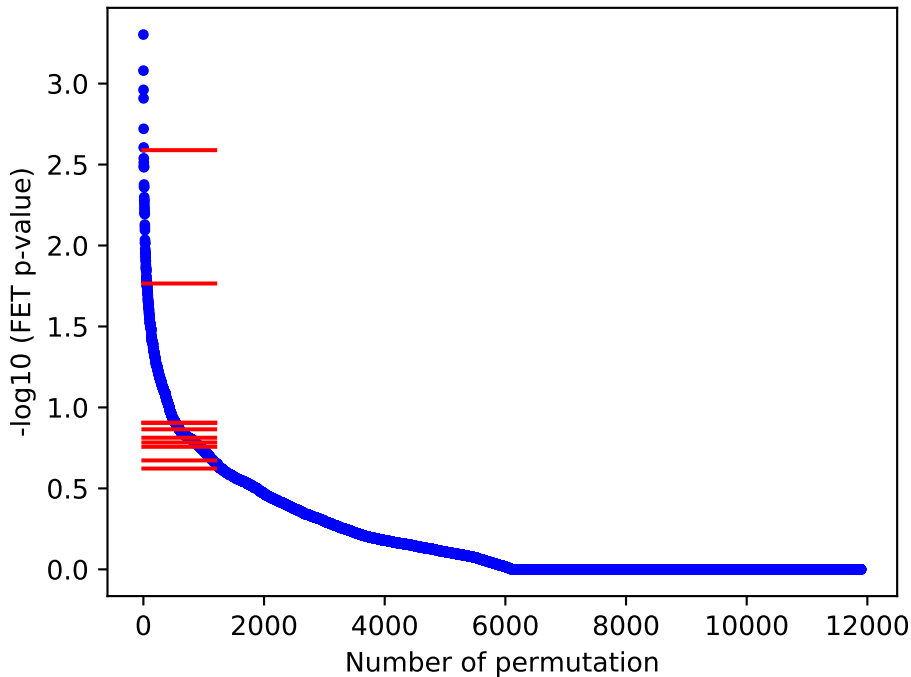

Supplement: Supplementary file 3 — Supporting information 3 [file 41366_2021_888_MOESM3_ESM.zip › figures/plot_pathwayModel_.pdf]

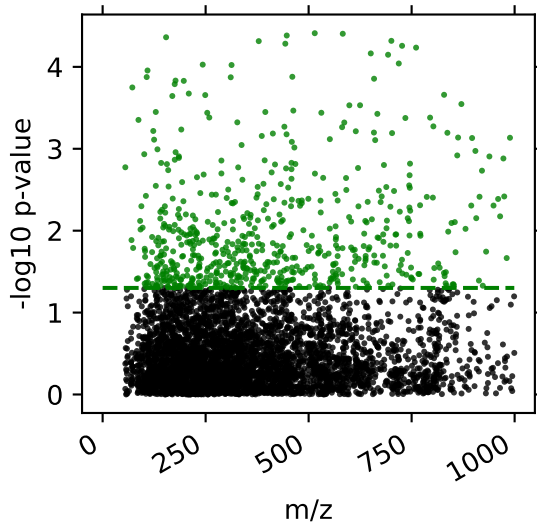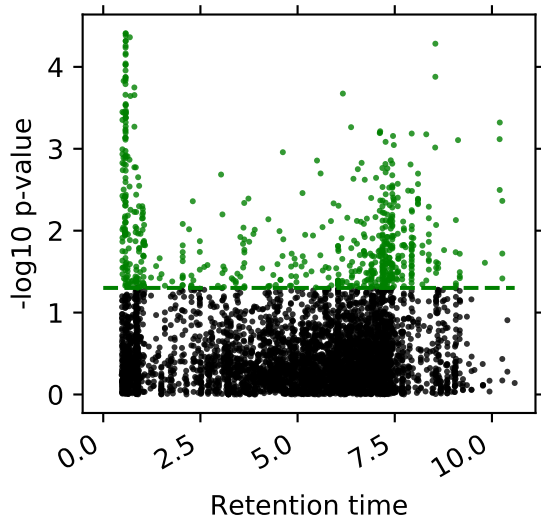

Supplement: Supplementary file 3 — Supporting information 3 [file 41366_2021_888_MOESM3_ESM.zip › figures/mcg_MWAS_.pdf]
